# The Mechanotransduction Signaling Pathways in the Regulation of Osteogenesis

**DOI:** 10.3390/ijms241814326

**Published:** 2023-09-20

**Authors:** Zhaoshuo Liu, Qilin Wang, Junyou Zhang, Sihan Qi, Yingying Duan, Chunyan Li

**Affiliations:** 1 School of Engineering Medicine, Beihang University, Beijing 100191, China; 2 School of Biological Science and Medical Engineering, Beihang University, Beijing 100191, China; 3 Key Laboratory of Big Data-Based Precision Medicine (Ministry of Industry and Information Technology), Beihang University, Beijing 100191, China; 4 Beijing Advanced Innovation Center for Big Data-Based Precision Medicine, Beihang University, Beijing 100191, China

**Keywords:** mechanotransduction, osteogenesis, microgravity, mechanical stimuli, bone formation

## Abstract

Bones are constantly exposed to mechanical forces from both muscles and Earth’s gravity to maintain bone homeostasis by stimulating bone formation. Mechanotransduction transforms external mechanical signals such as force, fluid flow shear, and gravity into intracellular responses to achieve force adaptation. However, the underlying molecular mechanisms on the conversion from mechanical signals into bone formation has not been completely defined yet. In the present review, we provide a comprehensive and systematic description of the mechanotransduction signaling pathways induced by mechanical stimuli during osteogenesis and address the different layers of interconnections between different signaling pathways. Further exploration of mechanotransduction would benefit patients with osteoporosis, including the aging population and postmenopausal women.

## 1. Introduction

Currently, a large number of elderly people, especially elderly women, suffer from osteoporosis. Of adults aged 50 years and above, 12.6% (19.6% in women and 4.4% in men) had osteoporosis at the femur neck, lumbar spine, or both in the USA in 2017–2018 [1]. Typical symptoms of osteoporosis include a decrease in bone mass and deterioration in the bone microstructure. Aging and menopause are the main risk factors for osteoporosis in women [2,3]. Estradiol deficiency increases the risk of osteoporosis in women during and after menopause [4,5]. In addition to estradiol deficiency, low mechanical load is another factor that increases the risk of osteoporosis. The occurrence of disuse osteoporosis can be attributed to the lack of mechanical loading on the skeletal system [6].

Bone mass loss occurs not only in individuals diagnosed with osteoporosis, but also in astronauts. Bone loss caused by microgravity was first observed in crews of the Skylab space station, which showed 1–2% decrease in bone mass every month [7,8,9]. A continuous decrease in bone density caused by space flight requires more time to recover to the pre-flight level [10,11]. Consistently, after seven days of space flight, mice showed 47–55% loss in tibial trabecular bone mass, 20–24% decrease in the thickness of trabecular bone, and 40–43% reduction in bone density [12]. Typical indicants of both osteoporosis and space bone loss include bone mass loss, decline in bone density, and structural impairment in bone tissue. Mechanical unloading is the primary factor contributing to disuse osteoporosis and space bone loss [6,13]. Therefore, studies on space bone loss due to microgravity will be inspiring to explore targets and therapeutic measures for osteoporosis. Nevertheless, due to the high expense and difficulties in space flight, there are limited numbers of space experiments on microgravity-induced bone loss.

Mechanotransduction is a process that includes a sense of the existence and changes in mechanical signals and signal transduction into the nucleus to regulate gene expression [14]. Here, we describe how microgravity affects osteogenesis by drawing mechanotransduction signaling pathways in detail and depicting the crosstalk between the pathways. A systemic overview on the mechanotransduction in osteoblast-lineage cells will provide clues to explore novel therapeutic targets against osteoporosis and bone loss during spaceflight.

## 2. Osteogenesis and the Differentiation of Osteoblast Lineage Cells

Osteogenesis, the process of bone development and formation, is crucial to maintain the skeletal system [15]. Osteogenesis is involved in the process of bone remodeling and the subsequent repair of bone fractures [16,17]. Osteogenesis is tightly regulated by mechanical and biochemical signaling pathways [18]. During osteogenesis, the sequential differentiation occurs in osteoblast lineage cells, including mesenchymal stem cells (MSCs), osteoprogenitors, pre-osteoblasts, osteoblasts, and osteocytes [16,17,19,20,21,22]. Osteoblasts secrete the organic matrix osteoid, which is subsequently calcified to form the bone. The calcification of osteoid is induced by the deposition of mineral salt. Subsequently, osteoblasts differentiate into osteocytes resulting in the formation of trabecular bone and periosteum [23].

**Figure 1:**
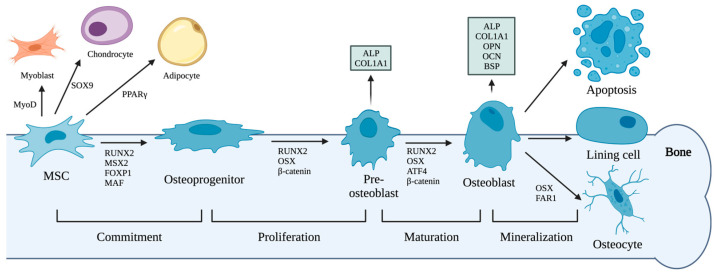
Schematic representation of the differentiation of osteoblast lineage cells. The differentiation process is divided into four stages. The representative markers for pre-osteoblasts and osteoblasts are presented in the corresponding boxes above the arrows, while TFs involved in the differentiation are presented beneath the arrows.

The differentiation of osteoblast lineage cells undergo four sequential stages: commitment, proliferation, maturation, and mineralization (Figure 1) [24]. MSCs have multiple differentiation fates, including osteoblasts, fibroblasts, adipocytes, and chondrocytes [25]. During the commitment stage, osteoprogenitor cells originate from MSCs and differentiate into osteoblasts [25,26]. Subsequently, the committed osteoprogenitors proliferate and differentiate into pre-osteoblasts [27]. Pre-osteoblasts express collagen type I alpha 1 (COL1A1) and alkaline phosphatase (ALP), which are necessary for the bone matrix formation and mineralization [28]. For mature osteoblasts, the expression of osteopontin (OPN), bone sialoprotein (BSP), and osteocalcin (OCN) are all elevated along with ALP and COL1A1 [24]. The glycoprotein ALP on cell surface hydrolyzes the mineralization inhibitor pyrophosphate into phosphate, which promotes the mineral deposition on the collagen fiber scaffold [29,30,31] OCN has high affinity to the hydroxyapatite matrix, and BSP enhances hydroxyapatite crystal formation [24]. OCN adjusts the alignment of apatite crystals parallel to collagen fibrils, to maintain bone mineral density and strength [32]. OPN inhibits hydroxyapatite formation, and decreases the differentiation of MSCs into osteoblasts [33,34]. After the matrix mineralization, mature osteoblasts undergo apoptosis, or form lining cells, or differentiate into osteocytes (Figure 1) [24,35]. Osteoblasts embedded in the mineralized bone matrix differentiate into osteocytes [36]. Non-apoptotic osteoblasts on bone surface become bone lining cells, which regulate bone remodeling by communicating with osteocytes [37].

The differentiation of osteoblast lineage cells is tightly regulated by transcription factors (TFs) (Figure 1) [38]. For MSCs, PPARγ, SOX9, and MyoD induce the differentiation into adipocytes, chondrocytes, and myoblasts, respectively [39,40,41]. Runt-related transcription factor 2 (RUNX2) is considered as the master switch for the initiation of osteogenesis, as RUNX2 is expressed in MSCs and further upregulated in pre-osteoblasts, while in osteoblasts, the expression of RUNX2 is decreased [42,43]. Osterix (OSX/SP7) induces the osteoblast differentiation, and inhibits the adipocyte differentiation [44]. Both RUNX2 and OSX induce the expression of ALP, OCN, OPN, BSP, and COL1A1 [24]. The co-activator β-catenin promotes the differentiation from pre-osteoblasts to osteoblasts [45]. Activating transcription factor 4 (ATF4), as one of the main transcription factors involved in osteoblast differentiation, functions via the upregulation of β-catenin [46,47,48]. TF Msh homeobox 2 (MSX2) promotes the osteogenic differentiation of MSCs and the calcification of osteoblasts [49]. Both AP1 protein FOS-related antigen 1 (FRA1) and JUNB, which are classic members of the Activator Protein 1 (AP1) transcription factor family, stimulate the osteoblast differentiation [50,51]. MAF bZIP transcription factor (MAF) positively regulates the osteogenic differentiation of MSCs, as well [52]. TF Forkhead box P1 (FOXP1) stimulates both the osteogenic differentiation of MSCs and the osteoblast mineralization [53,54].

Among multiple TFs regulating osteoblast differentiation, RUNX2 and OSX (encoded by gene *SP7*) are master TFs in the differentiation from MSCs into osteoblasts [55]. Therefore, expressions of *RUNX2* and *SP7* are commonly used as markers of osteogenic differentiation of MSCs. In addition to TFs, there are multiple makers characterized for different stages of osteoblast differentiation. As the contact between osteoblasts and collagen type I is essential for the differentiation of osteoblasts, collagen type I, especially COL1A1, is another maker for the differentiation from MSCs into osteoblasts [56,57]. As ALP enhances mineralization of ECM, the quantification of ALP at both mRNA and protein levels has been used to describe the differentiation of osteoblasts [28]. BSP, OPN, and OCN, which promote mineralization of ECM, are used as makers of differentiation of mature osteoblasts [58,59].

## 3. Mechanotransduction and Osteogenesis

### 3.1. Mechanical Stimuli and Osteogenesis

Both osteogenesis and the differentiation of osteoblast lineage cells are regulated by alterations of mechanical stimuli, such as microgravity. Microgravity simulation inhibits the osteogenic differentiation of MSCs and the process of mineralization, but promotes the adipogenic differentiation [60]. After 24 h of microgravity simulation, the mRNA levels of *Alp*, *Ocn* (osteocalcin), and *Runx2* in osteoblasts decreased by approximately 80%, 50%, and 60%, respectively [61]. After seven days of microgravity simulation, the activity of Alp was significantly reduced, and the expression of osteoblastic differentiation genes, including *Ocn*, *Col1a1*, and *Runx2*, was downregulated [62]. To dissect the underlying mechanisms between bone loss and microgravity, it will be helpful to explore new strategies or therapeutic targets to alleviate osteoporosis and space bone loss.

**Figure 2:**
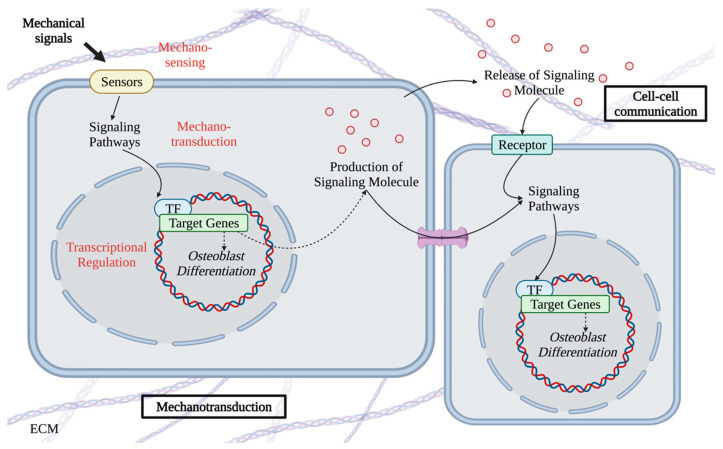
The mechanotransduction within or between bone cells. The signaling molecules transmit the mechanical signals into cells or nearby cells. The red fonts highlight the three sequential steps of mechanotransduction.

Mechanical stimuli directly affect the differentiation of MSCs and osteoblasts in vitro, whereas osteocytes are the major mechanosensitive sensors in bone tissue. Via mechanotransduction, mechanical signals regulate the osteogenic gene expressions and the release of signaling molecules. Subsequently, osteocytes influenced by mechanical signals regulated MSCs and osteoblasts. The lacunar canalicular system (LCS) is the fundamental structure for mechanosensing in osteocytes. The lacunae outside the cell body of osteocytes together with the tubules outside the dendrites of osteocytes form the LCS [63]. Both the osteocyte network and LCS are essential for the functions of osteocytes. Between the mineralized matrix and osteocytes, there is a 50–100 nm lacunae [64]. The tissue fluid fills the gaps within the collagen fiber layer [64]. Osteocytes, which are the primary cells responding to mechanical stimuli, are regulated by fluid shear stress under normal physiological conditions [65]. Mechanical unloading by microgravity during space flight induces bone matrix resorption and rebuilding around osteocytes [66,67]. Osteocytes transmit mechanical signals from the external environment to osteoblasts and osteoclasts by releasing molecules including ATP, prostaglandin E2 (PGE_2_), nitric oxide (NO), and growth factors [68,69,70,71]. Once osteocytes sense mechanical signals, mechanotransduction is initiated to induce the production and release of signaling molecules, such as ATP and PGE_2_. Signaling molecules activate intracellular pathways in osteoblasts by binding to the receptors on cell membrane or by translocating into cells via channels. Ultimately, the activities of osteoblasts, such as differentiation, are altered in response to the mechanical signals, via both intracellular mechanotransduction and cell-cell communications (Figure 2). Generally speaking, the process of mechanotransduction includes three sequential steps: mechanosensing, mechanotransduction pathways, and transcriptional regulation.

### 3.2. Mechanosensing on the Cell Membrane

Cells respond to mechanical signals, including physical forces, ultrasonic waves, and electromagnetic waves, to adapt to environmental changes. Since mechanical load stimulates bone growth, there have been numerous studies on the mechanisms of mechanosensing, the first step of mechanotransduction [72]. Mechanosensing is the process including the altered structure of sensors and transmission of extracellular signal into intracellular pathways [73]. Integrins, Piezo channels, primary cilia, and gap junction (GJ)-mediated mechanosensing have been extensively studied. In response to mechanical stimuli, integrin proteins change their structure to induce the formation of focal adhesions (FAs). Mechanosensitive calcium channels, represented by the Piezo calcium channel protein family, switch from closed to open in response to mechanical stimuli, resulting in extracellular calcium ion influx and a series of biochemical reactions. Activated by fluid flow shear stress, the cAMP level recedes in primary cilia. Mechanical stimuli switch GJs to the open state to allow signaling molecules, including calcium ions, ATP, and PGE_2_, to translocate into neighbor cells. Signaling molecules translocate to ECM via GJs, as well. The mechanical sensors, including integrins, Ca^2+^ channels, and GJs, will be introduced one-by-one in detail.

#### 3.2.1. Integrins Sense Mechanical Signals through Integrin-ECM Interaction

Integrins, a family of transmembrane proteins, include non-covalently linked α and β subunits. Integrins function as receptors in heterodimer to initiate the assembly of signaling complexes, which subsequently link ECM to cytoskeleton [74]. The integrin heterodimers α1β1, α2β1, α5β1, α6β1, αVβ3, and αVβ5 have been characterized in human MSCs (hMSCs) [75]. The α1, α3, and β1 integrin subunits are predominantly expressed, whereas α2 is weakly expressed in osteoblasts [76]. Integrin α1-5, αV, β1, and β3 are expressed in osteocytes [77,78]. Since the abnormality in integrin is associated with bone diseases, integrins are potential therapeutic targets for bone loss. For example, the impairment of αVβ3 integrins in osteocytes results in the attenuation of mechanosensing [78].

ECM, as a three-dimensional non-cellular macromolecular network, is composed of collagens, proteoglycans, and glycosaminoglycans, such as elastin, fibronectin, and laminins [79]. Type I, III, and V collagen proteins, the most abundant constituents of ECM in bones, mainly act as scaffolds for mechanical support in bone cells [80]. Small leucine-rich proteoglycans interact with the collagen framework, cytokines, and receptors to regulate the proliferation, differentiation, and especially mineralization [81]. As an essential ECM component, BSP, a heavily glycosylated and phosphorylated protein, promotes osteoblast differentiation and initiates matrix mineralization [82].

**Figure 3:**
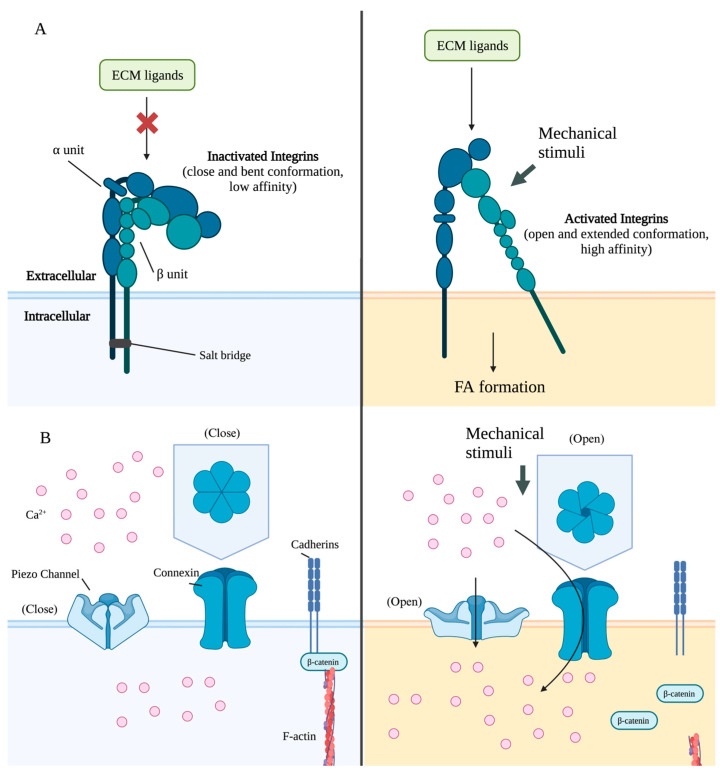
The activation of sensors by mechanical stimuli. (**A**) The conformation of integrins switches from the close and bent conformation (**left**) to the open and extended conformation (**right**) by mechanical stimuli. The activated integrins initiate the FA formation for mechanotransduction. (**B**) Mechanical stimuli transform channel proteins, including Piezo and connexin 43, from closed status (**left**) to open status (**right**). β-catenin combined with cadherins in a static state (**left**), but are released into cytoplasm with mechanical stimuli (**right**).

The mechanotransduction is initiated by the interactions between integrins and ECM [83]. Integrin heterodimers are inactivated by the cytoplasmic salt bridges. When integrins are inactivated, the extracellular domains are in a close and bent conformation with a low ligand-binding affinity (Figure 3A) [84]. Mechanical forces stretch the integrin binding sites on cell surfaces to switch them into an open and extended conformation, with high affinity to ECM ligands, such as laminin (ligand for α6β1), fibronectin (ligand for α5β1and αVβ3), or vitronectin (ligand for αVβ3) (Figure 3A) [84,85,86]. The activated integrins then aggregate and reinforce the connections between cells and ECM [87,88].

A series of integrins, including α5β1 and α3β1, mediate the direct interactions between osteoblast-lineage cells and ECM. For example, specific α5β1 integrins interact with fibronectin, which is a type of glycoprotein distributed ubiquitously in ECM during osteogenic differentiation [89,90]. Under Rotating Cell Culture System (RCCS) microgravity simulation for seven days, the differentiation of hMSCs into osteoblasts was severely impaired, the expression of the ECM proteins and type I collagen (Col I) was decreased, and the expression of Col I-specific α2 and β1 integrin proteins was enhanced [91]. However, FA formation and the activation of FA signal proteins were subsequently decreased. The activation of downstream pathways is influenced by the alterations in ligand accessibility and the quantity of integrins [91]. Above all, integrins are involved in mechanotransduction in response to mechanical changes.

#### 3.2.2. Ca^2+^ Channels as Mechanical Sensors

Calcium ion channels transform extracellular mechanical signals into intracellular biochemical signals [92,93]. Calcium intake benefits bone health via the regulation on cell-cell communications and cell-ECM interactions [94,95]. Intracellular calcium signaling is widely involved in the regulation of cell proliferation, differentiation, and metabolism [96].

Piezo proteins, including Piezo1 and Piezo2, were identified as mechanosensitive Ca^2+^ channels in 2010 [97]. Using cryoelectron microscopy, the structures of mouse Piezo1 and Piezo2 have been depicted in detail [98,99]. Piezo1 is a three-bladed propeller-like homotrimer with two modules: a central ion-conduction pore module and a peripheral mechanotransduction module [100,101]. And the structures and functions of Piezo1 and Piezo2 proteins are highly similar.

Piezo proteins play important roles in osteogenic differentiation. In MSCs, Piezo1 senses hydrostatic pressure to promote osteogenic differentiation and inhibits differentiation into adipocytes [102]. *Piezo1* knockout results in bone formation failure and stunted bone in adulthood, and consistently, the decrease of Piezo1 channels is one of the causes of osteoporosis [103]. Fluid shear stress upregulates the expression of *Piezo1* and induces the expression of *Runx2* in pre-osteoblasts MC3T3-E1 cells [104]. As a type of mechanical stimuli, low-intensity pulsed ultrasound (LIPUS) transduced by Piezo1 increases the concentration of intracellular calcium [105]. Mechanical signals induce cell membrane deformation, and the tension from the deformed cell membrane stretches the Piezo1 channel to open the central pore module (Figure 3B) [106,107]. Extracellular cations, including calcium ions, enter cells through Piezo channels to activate downstream pathways [108,109]. The microgravity simulation significantly reduced the expression of *Piezo1* and *Alp*, whereas fluid shear stress upregulated their expression [103].

The primary cilium, a non-motile microtubule-based organelle protruding from cell surface, senses extracellular chemical and mechanical signals and transduces mechanical signals into cells [110,111]. Primary cilia are in hMSCs, osteoblasts, and osteocytes [111,112,113]. When cells are exposed to fluid flow shear, the primary cilia deflect and recoil accordingly with fluid flow [114]. Using siRNAs to inhibit Polaris, a protein necessary to the primary cilia formation, resulted in the transcriptional reduction of *RUNX2* and *PPARG* [115]. The expression level of *OPN* was increased in osteoblasts 1 h after exposure to oscillatory fluid flow [111]. The removal of primary cilia by chloral hydrate treatment via the disruption of the connection between primary cilia and the basal body, results in the failure of response to fluid flow for osteoblasts. The mRNA expression of *OPN* is increased by fluid flow [116]. However, the transcriptional increase of *OPN* is damaged by the removal of primary cilia [111]. Therefore, without primary cilia, cells fail to respond to fluid flow.

The mechanosensitive calcium channel TRPV4 in primary cilia is a member of the transient receptor potential (TRP) channel family [117]. TRPV4, 871 amino acids with six transmembrane α-helices, is expressed in MSCs, osteoblasts, and osteocytes [118]. TRPV4 regulates bone homeostasis by controlling the Ca^2+^ influx [119,120,121,122,123]. Dominant mutations in *TRPV4* cause several types of skeletal dysplasia, including metatropic dysplasia, spondylometaphyseal dysplasia Kozlowski type (SMDK), and autosomal dominant brachyolmia [124]. Mechanical forces promote osteoblastic differentiation by enhancing the expression of *TRPV4* [125,126]. The primary cilia-dependent calcium ion channel TRPV4 mediates fluid shear signal transduction by inducing calcium ion into primary cilia. Even when TRPV4 is activated, the osteogenic differentiations of MSCs with defective primary cilia are inhibited [127]. Therefore, primary cilia and TRPV4 channels collaborate to sense mechanical signals.

#### 3.2.3. Gap Junctions and Bone Cell Communication

Both GJs and hemichannels, which are composed of connexins, are involved in the transmission of mechanical signals. The hexameric connexin protein is composed of six homogenous or heterogeneous connexin subunits. Hemichannels provide communication between the intracellular cytoplasm and extracellular ECM [128]. GJs between adjacent cells enable the communication of neighboring cells [129]. For example, GJs between osteoblasts and MSCs promote the transcription of *ALP* in MSCs [130]. Connexin 43, expressed in osteocytes, osteoblasts, and MSCs, is responsible for the gap junction formation [131].

Mechanical stimuli are transmitted via altering the expression of connexin 43 and status of GJs. In bone tissue, signaling molecules, including PGE_2_, ATP, cAMP, and Ca^2+^, diffuse from cells via GJs or hemichannels to stimulate signaling pathways in neighbor cells [132,133,134,135]. The expression of connexin 43 was increased in MC3T3-E1 cells after 5 h treatment with microstrain [136]. The mechanical signal transforms GJs and hemichannels from closed to open status to exchange signaling molecules (Figure 3B) [77]. Response to fluid shear stress, PGE_2_, and ATP are released via hemichannels from osteocytes into ECM [132,133].

In addition to GJs, cadherins implement the communication between nearby cells. In osteoblast lineage cells, N-cadherin (cadherin-2, CDH2) and cadherin-11 (CDH11) are predominantly expressed [137]. The expression of cadherins, including *Cdh2* and *Cdh11*, is low in MSCs, while the expression of *Cdh2* and *Cdh11* is increased with the differentiation commitment to the pre-osteoblasts [138]. During the progress of the osteoblast differentiation, *Cdh2* is downregulated and subsequently rarely expressed in mature osteoblasts, but *Cdh11* is present throughout the osteoblast differentiation [138]. The adhesion mediated by cadherin is crucial for the early stage of osteoblast differentiation [139]. The function loss of cadherin inhibits osteoblast differentiation, but enhances adipogenic differentiation [140]. Cadherins are essential to osteogenesis, as there are developmental defects and low bone mass in *Cdh2* mutant mice [138].

As a type of sensor, cadherins mediate the transmission of mechanical stimuli. In a static state, β-catenin mediates the connections between cadherins on cell membrane and cytoplasmic cytoskeleton. In response to mechanical stimuli, such as fluid flow shear, the release of β-catenin from cadherins and the β-catenin accumulates in the cytoplasm subsequently (Figure 3B). The cytoplasmic β-catenin undergoes translocation into the nucleus and triggers transcription in response to mechanical stimuli [141,142].

### 3.3. The Cytoplasmic Mechanotransduction Pathways

For the pathways involved in mechanotransduction during osteogenesis, both the integrin-focal adhesion (FA)-cytoskeleton pathway and the RhoA pathway depend on the activation of integrins. Both the NFAT-Ppp3Ca pathway downstream of Piezo and mitogen-activated kinase (MAPK) pathways depend on the influx of calcium ions. The decrease in cAMP level in primary cilia enhances the promotion of osteogenesis by cyclooxygenase-2 (COX-2) and inhibits the activity of β-catenin by regulating its activation or the translocation into the nucleus.

#### 3.3.1. Cytoskeleton Reorganization by FA and RhoA Pathway

The cytoskeleton, as a prestressed tensegrity structure, receives and sustains force, stabilizes cells, and facilitates cells to adapt to environmental alterations [143]. Filamentous actin (F-actin), as the main form of cytoskeleton, is a polar polymer of globular actin (G-actin). At the barbed and pointed end of F-actin, G-actin is polymerized and de-polymerized, respectively. The binding of cofilin induces the severing of F-actin [144]. The reorganization of the actin cytoskeleton is a response to mechanical forces. Actin filaments are extended and stabilized in a direction parallel to the force, and then myosin II replaces cofilin to associate with actin. Microtubules (MTs) and intermediate filaments (IFs), other types of cytoskeletons, are involved in mechanotransduction, as well. MTs are acetylated by integrin-mediated substrate-rigidity sensing [145]. IFs are crucial in the regulation of cell shape and maintenance of mechanical integrity [146].

**Figure 4:**
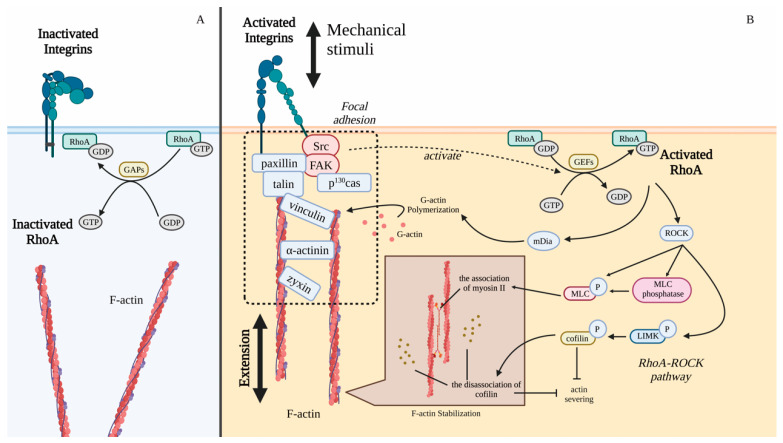
The activated integrin recruits signal proteins and structural proteins to form FA to respond to mechanical stimuli. (**A**) Without mechanical stimuli, RhoA is inactive as RhoA-GDP. (**B**) Mechanical stimuli activate integrins to initiate FA formation. Through FAK-Src complex, RhoA pathway is activated by integrin-mediated mechanical stimuli. mDia activated by RhoA promotes the polymerization of G-actin. ROCK activated by RhoA promotes the dephosphorylation of MLC and cofilin to stabilize the cytoskeleton. Integrin-FA complex reorganizes the actin cytoskeleton and transmits the extracellular mechanical signals into cells.

The activated integrins mediate the formation of focal adhesions (FAs), which connect integrins to the F-actin cytoskeleton. FAs are composed of signal proteins and structural proteins. Signal proteins, including Src and focal adhesion kinase (FAK), are crucial for mechanical transmission [147]. Once the external mechanical stimuli are sensed, FAK is recruited to FA first, and the structural proteins, including talin, paxilin, vinculin, and zyxin, are recruited to the complex, subsequently [147,148,149,150,151]. The binding between vinculin and talin further stabilizes the interaction between talin and F-actin, and thus transfers the mechanical signal inward [152]. The structural protein p^130^Cas is phosphorylated by the FAK-Src complex to respond to mechanical stress, such as the attachment and spreading [153,154,155]. The connection between integrin and actin mediated by FA reorganizes cytoskeleton to adapt to environmental changes, such as mechanical stimuli (Figure 4).

RhoA signaling is involved in mechanotransduction via the regulation of cytoskeletal stabilization [156]. FAs mediate mechanical signals, including fluid shear stress, from activated integrins to RhoA [157,158,159]. After the sensing of external mechanical stimuli, FAK and Src are recruited and activated, which subsequently activate RhoA. RhoA, a GTPase, regulates various cellular activities, including actomyosin dynamics, adhesion, proliferation, and survival [160]. The interaction between GDP and RhoA is essential to maintain RhoA in an inactive state in the cytoplasm [161]. GTPase-activating proteins (GAPs) transform RhoA into an inactive state by converting GTP to GDP (Figure 4A) [162]. In contrast, RhoA is activated by guanine nucleotide exchange factors (GEFs) by catalyzing GDP to GTP (Figure 4B) [163].

The stabilization of the actin cytoskeleton is enhanced via the actin polymerization and the F-actin-severing inhibition by the RhoA pathway. Both mDia and ROCK are downstream effectors of the activated RhoA (Figure 4B) [164,165]. The effector mDia promotes the extension of F-actin through enhancing the polymerization of G-actin [166]. Another effector ROCK alters the association between F-actin of myosin II and cofilin. ROCK increases the phosphorylation of myosin light chain (MLC) to promote the assembly of myosin II into bipolar filaments, and inhibits the dephosphorylation of phosphorylated MLC [167,168]. The ATPase activity of myosin II is enhanced by ROCK. The association between myosin II and the phosphorylated MLC further increases the stability of actin. LIM kinase (LIMK) is phosphorylated by ROCK. The activated LIMK inactivates cofilin, which is an F-actin-severing protein [169]. The dissociation of cofilin from actin results in the inhibition of F-actin severing (Figure 4B).

Cell density alters cell shapes and drives hMSC commitment via the activation of RhoA, which subsequently regulates ROCK and cytoskeletal integrity [170]. Under enhanced or minimized mechanical stimuli, such as forces or microgravity, cell shape will be changed [171,172]. The intracellular cytoskeleton is resistant to the deformation induced by the extracellular mechanical stimuli, as the cytoskeleton is highly dynamic and adaptive [173]. Similarly, in response to a reduced gravitational load, from 1 *G* to microgravity, cells change cytoskeletal structures accordingly [174,175]. The reorganization of actin filaments in simulated microgravity provides clues of the altered cytoskeleton function in mechanotransduction [176].

#### 3.3.2. Downstream Pathways of Ca^2+^

**Figure 5:**
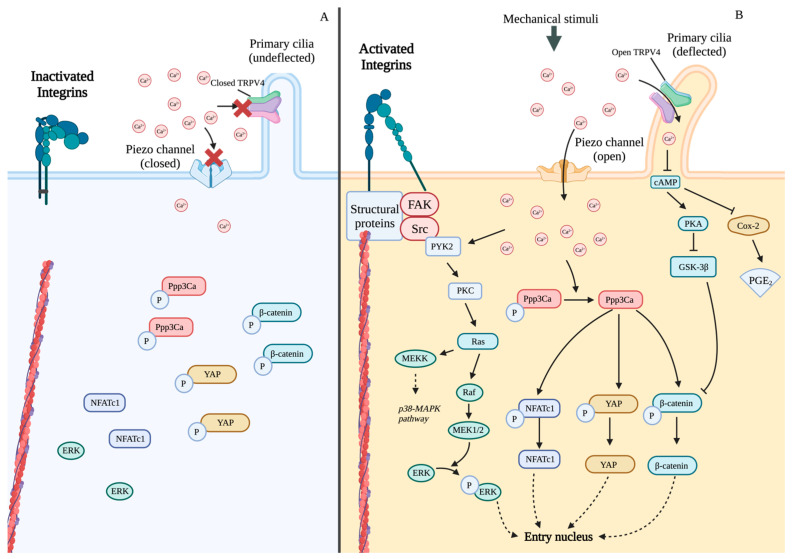
Mechanosensitive Ca^2+^ channels are open with mechanical signals, and subsequent Ca^2+^ influx initiates downstream pathways. (**A**) The mechanosensitive Ca^2+^ channels are close in static environment. (**B**) The mechanical stimuli switch the mechanical-sensitive Ca^2+^ channels to the open state. The influx of Ca^2+^ activates multiple pathways. In deflected primary cilia, influx of Ca^2+^ results in the reduction of cAMP.

The open Piezo channel activates the downstream pathways using calcium ions as second messengers. Calcium ions were first characterized as second messengers in the excitation-contraction coupling in skeletal muscles [177]. Calcium influx through the Piezo channel in osteoblasts promotes the phosphorylation of ERK1/2 and the polymerization of perinuclear F-actin filaments [105]. In addition, calcium influx through the open Piezo channels induced by mechanical signals activates calmodulin-dependent heterodimer serine/threonine phosphatase calcineurin (Ppp3ca). Nuclear factor of activated T cells (NFAT), Yes1 associated transcriptional regulator (YAP1), and β-catenin are activated via Ca^2+^/Ppp3ca activated by Piezo1 (Figure 5) [178]. Nuclear NFAT and SP7 (Osterix) form transcriptional complexes to trigger the expression of osteogenic genes, including *COL1A1*, *ALP*, *SPP1(OPN)*, and *BGLAP (OCN)* [179,180]. The activated calmodulin-calcineurin pathway dephosphorylates NFAT in osteoblasts [181].

Calcium influx induced by mechanical signaling activates Runx2 via the Ras/ERK-MAPK pathway, a subfamily of MAPK pathway families (Figure 5) [182,183,184]. The activation of the MAPK pathway by tensile and shear stress, hence, facilitates the transmission of mechanical signals [185]. Via the ERK-MAPK pathway, mechanical stress promotes osteogenic differentiation and osteogenesis [182,183]. The phosphorylated ERK activated by the MAPK pathway is translocated into the nucleus to phosphorylate RUNX2, which decompresses the chromosome and promotes the transcription of osteogenic genes [186].

The deflection of primary cilia caused by fluid shear activates Ca^2+^ channels, and intracellular Ca^2+^ influx inhibits the activity of adenylyl cyclase 6 (AC6), resulting in the decreased levels of cAMP [115]. The drop in cAMP in the primary ciliary activates osteogenesis by promoting the expression of COX-2, which produces PGE_2_ and further regulates RUNX2 and SP7 to mediate osteogenesis and bone repair (Figure 5) [187,188]. Protein kinase A (PKA), another downstream effector in primary cilia, activates the ERK1/2-CREB signaling pathway and inhibits glycogen synthase kinase 3 β (GSK3β)-mediated degradation of β-catenin [116,141,189]. Through the MAPK and Ras/Raf-dependent ERK1/2 pathways, mechanical stress increases the activated Runx2 and promotes osteoblast differentiation.

### 3.4. Nuclear Alterations and the Transcriptional Regulation

**Figure 6:**
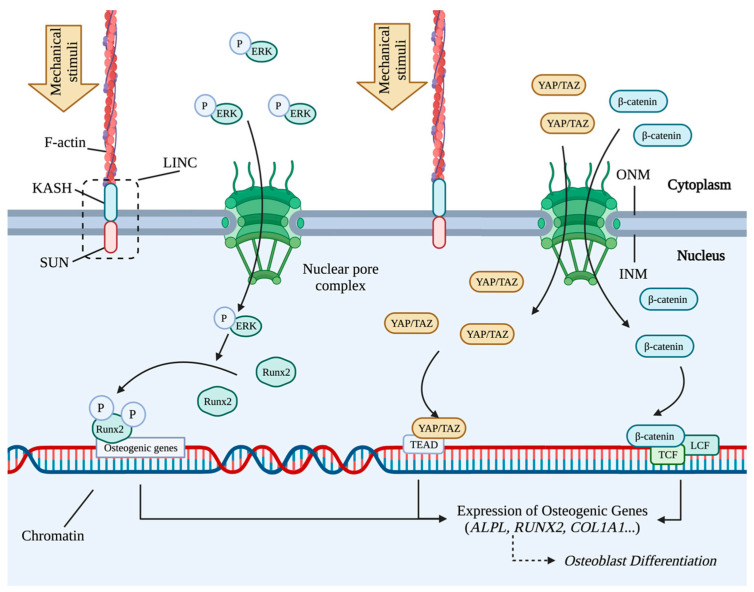
The responsive translocation of TFs and the transcriptional regulation by mechanotransduction during osteogenesis. Mechanical stimuli are transformed to nuclear stretch via the interactions between LINC and F-actin. Subsequently, NPCs on nuclear membrane are allowed increasing TFs and coactivators to enter into the nucleus. More YAP/TAZ and β-catenin proteins enter nucleus and promote the expression of osteogenic genes. The binding by the phosphorylated Runx2 transforms the nearby chromosomal region to open status and stimulates the expression of osteogenic genes.

For the transmission of mechanical signals into the nuclei, the linker of the nucleoskeleton and cytoskeleton (LINC) is necessary to connect the nucleoskeleton, nuclear envelope, and cytoskeleton [190]. Inner SUN and outer KASH domain proteins form the core of the LINC complex [191]. The nuclear envelope consists of inner and outer nuclear membranes (INM and ONM), where SUN and KASH anchor (Figure 6) [192]. By the interaction with LINC and F-actin, the nucleus acts as a mechanical-sensitive subcellular compartment [193]. The alteration in nucleus shape, induced by cell deformation and medicated by cytoskeleton-LINC, influences the intranuclear transcription. Under a 1 *G* gravity environment, the nucleus has a large, round shape, but microgravity makes the nucleus 30% smaller [194]. Nuclear pore complexes (NPCs) on the nuclear membrane mediate the transport between the cytoplasm and the nucleus [195]. Stimulated by mechanical force, the permeability of nuclear pores is increased by the LINC-regulated nuclear stretch, and more TF proteins enter the nucleus subsequently (Figure 6) [196].

In the nucleus, RUNX2, YAP, TAZ, and β-catenin are responsive to mechanical stimuli. In the ERK-MAPK pathway, Runx2 is activated and transmits the mechanical signals to gene expression regulation (Figure 6). In the nucleus, the phosphorylated ERK of the MAPK pathway phosphorylates RUNX2 protein to enhance the binding between the histone acetyltransferase p300 to achieve the acetylation of histone via H3K9ac and H4K5ac [186]. The epigenetic changes decondense chromatin and increase the transcription of osteogenic genes via the recruitment of RNA polymerase II [186].

As both YAP and TAZ lack DNA-binding domains, YAP and TAZ must bind to coactivators to activate the transcription of target genes [197]. Both in vitro and in vivo experiments showed that the inhibition on the interaction between YAP/TAZ and the transcriptional enhanced associate domain (TEAD) reduced the expression of osteogenic genes (Figure 6) [198]. Compared to the control group, in YAP or TAZ knockout mice, matrix collagen contents were reduced and bone microstructures were damaged [198]. YAP promotes osteogenesis and suppresses adipogenesis by interacting with and stabilizing β-catenin protein to maintain the nuclear level of β-catenin [199]. TAZ stimulates osteoblast differentiation via activating Runx2 [200]. YAP/TAZ is involved in diverse steps of osteogenesis; for example, YAP/TAZ inhibits MSC differentiation into osteoblasts, promotes bone formation, and inhibits bone resorption in mature osteoblasts and osteocytes [201].

β-catenin, encoded by *CTNNB1*, is a multifunctional protein [202,203]. β-catenin shuttles between the cytoplasm and nucleus [204]. Cytosolic stable β-catenin enters the nucleus to bind to T-cell factor/lymphoid enhancer-binding factor (TCF/LEF) proteins and activate transcription subsequently, including osteogenic gene expressions (Figure 6) [202,205]. In bone tissue, β-catenin promotes bone formation and inhibits bone resorption in both mice and human [206,207]. β-catenin stimulates the differentiation of pre-osteoblasts into osteoblasts [46].

### 3.5. Crosstalks during Mechanotransduction

Mechanotransduction is summarized as three sequential steps as above: mechanical sensors on cell membrane, cytoplasmic mechanotransduction pathways, and transcriptional regulation. There are crosstalks at each layer, which make the mechanotransduction an interconnected network.

**Figure 7:**
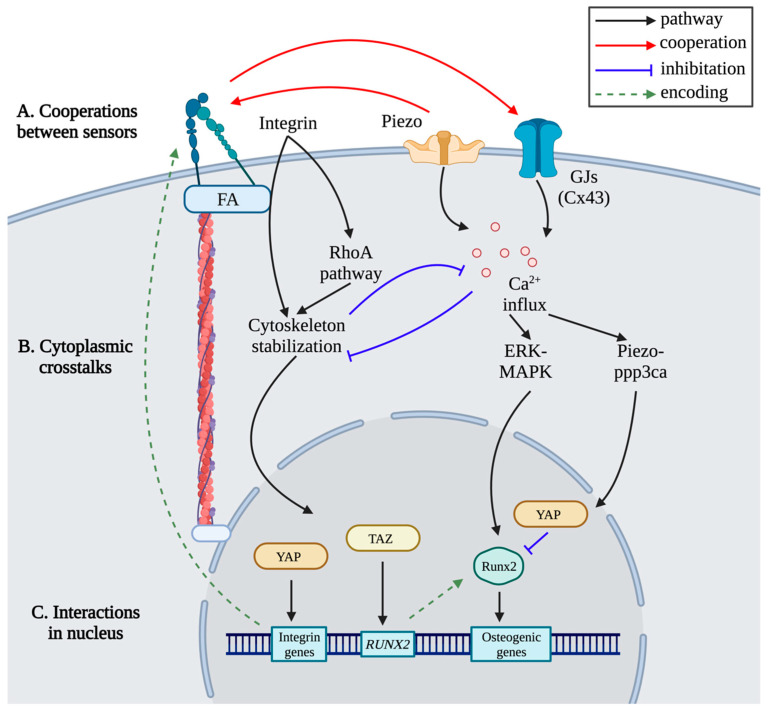
Crosstalks during mechanotransduction. In mechanotransduction, sensors cooperate with each other. Within the cytoplasm, cytoskeleton and Ca^2+^-involved pathways have crosstalks. The expression of osteogenic genes is coordinated by different TFs and coactivators, including *RUNX2* and integrin protein coding genes.

The cooperation between different sensors enhances the mechanotransduction initiation (Figure 7A). For osteocytes, fluid shear stress stimulated sensors connexin 43 hemichannels via activating αV and α5 integrins [208]. Sensor Piezo1 binds to integrins and promotes the formation of FA [209]. Fluid shear stress leads to Piezo1-mediated integrin activation resulting in FAK activation [210].

Cytoskeleton and cytoplasmic Ca^2+^-mediated pathways are responsible for the transformation of mechanical stimuli from sensors to nuclear. In mechanotransduction pathway networks, cytoskeleton and cytoplasmic Ca^2+^ are central components. Cytoskeleton and Ca^2+^ interact with each other (Figure 7B). The increase in calcium inhibits the elongation of filaments [211]. In osteoblasts, the polymerization of actin increases the influx of calcium ions, while depolymerization decreases the influx [212]. After the sensing of mechanical signals, cytoskeletal mechanical sensors activate mechanical transducers including Ca^2+^ influx [213].

The expressions of osteogenic genes are regulated by intranuclear interactions (Figure 7C). TAZ coactivates the transcription of RUNX2-dependent genes, and promotes the osteogenic differentiation of MSCs [214]. Interactions between YAP and RUNX2 suppress the transcriptional activity of RUNX2 [215]. As the *Runx2* promoter region has TCF response elements, β-catenin positively regulates Runx2 expression [216]. In addition, TFs and coactivators affect sensors and components of pathways in mechanotransduction via expression regulation. For example, integrin genes *ITGA1*, *ITGA4*, and *ITGAV* are target genes of YAP [217].

## 4. Perspectives and Conclusions

Osteogenesis plays a crucial role in the maintenance of bone mass and strength. Mechanical signals influence osteogenesis via mechanotransduction, which is the process that transmits mechanical signals to the nucleus to regulate gene expression. Further investigation on mechanotransduction will potentially provide a comprehensive understanding of the molecular mechanisms of bone loss and facilitate the development of a therapeutic strategy against osteoporosis, as well.

The principles of osteoporosis therapy are to improve bone formation and/or to decrease bone resorption. Bisphosphonates inhibit bone resorption via the inhibition on the activity of osteoclasts [218,219]. Bisphosphonates are widely prescribed in clinical practice, including alendronate, risedronate, ibandronate, and zoledronic acid [220,221,222,223]. Among multiple bisphosphonates, alendronate has been proven to alleviate bone loss of the astronauts on long-term space missions [224]. It is convenient to take bisphosphonates orally, but bisphosphonates are commonly employed for a maximum duration of 10 years in the long-term management of osteoporosis [225,226]. In addition, bisphosphonates are associated with adverse effects, such as osteonecrosis of the jaw, delayed dental eruption, atypical femoral fracture, and ocular side effects [227,228]. Another commonly prescribed anti-bone resorption drug, Denosumab, is more effective against bone loss than bisphosphonate [229]. But denosumab increases the incidence of adverse events, including hypocalcemia, osteonecrosis of the jaw, and atypical fractures [230]. From the perspective of bone formation, bone-building drugs have been developed to alleviate osteoporosis, and teriparatide, abaloparatide, and romosozumab are commonly recommended [231,232,233]. In comparison to anti-bone resorption drugs, bone-building medications are more efficient to enhance bone mass density and have fewer side effects [234,235]. Bone-building medications are appropriate for patients experiencing complications from bisphosphonate treatment, but the maximum usage lasts for only 2 years [236,237]. And the subcutaneous injection makes bone-building medications less convenient to take [232,238]. As bone mass was increased by bone-building medications in mice models under microgravity simulation, it is likely that the bone-building medications will bring benefits to astronauts against space bone loss [239]. However, the hypothesis remains to be validated by experiments in orbit. Since both bisphosphonates and bone-building medications have limitations, it is demanding to explore pharmaceuticals against bone loss with less adverse effects and more convenience.

In addition to pharmacological interventions, mechanical stimuli represent an alternative therapeutic approach for mitigating bone loss by promoting bone growth. LIPUS, as a type of mechanical stimulus, has significant advantages for osteogenesis by promoting the differentiation of osteoblasts, thereby effectively facilitating bone regeneration [240]. In 1994, LIPUS was approved as an adjuvant therapy in the healing of primary fractures [241]. In Canada and the UK, LIPUS is available for patients as a prescribed treatment [242]. An extremely low-frequency pulsed electromagnetic field, which is a type of mechanical stimuli, enhances the proliferation and differentiation of osteoblasts [243]. The utilization of mechanical stimulation is associated with minimal side effects, mostly due to the non-invasiveness. Studies on mechanotransduction may provide new potential therapeutic targets by mechanical stimulation to cure osteoporosis.

Further investigation on the molecular basis of mechanotransduction in bone physiology is essential to explore mechanically oriented therapeutic strategies. The components of mechanotransduction are potential targets that facilitate the responses to mechanical loading. Previous research has demonstrated that the activation of integrin αV, a sensor involved in mechanotransduction, represents a potential for the treatment of osteoporosis [244]. Delivery strategies have been explored to target bone cells, specifically MSCs and osteoblasts. Lipid nanoparticles and liposomal transport have been employed for the targeted delivery of medicines into osteoblasts and MSCs, as documented in previous studies [245,246]. A bone-targeting technology has been developed for the delivery of siRNA, and the efficacy has been evaluated in a preclinical investigation [247,248]. In mice, exosomes deliver S8178, a Wnt agonist, to bone specifically, thereby facilitating the osteogenic differentiation of MSCs [249]. Bisphosphonates, which have an affinity to hydroxyapatite, are applied to implement bone tissue-specific targeting [246]. By integrating iron oxide nanoparticles and bisphosphonates, the treatment of osteoporosis was efficient by increasing the bone mechanical strength [250].

Above all, this review provides a comprehensive overview of mechanotransduction during osteogenesis. The mechanotransduction pathways and crosstalks described in this review provide potential targets against bone loss. Among the components of mechanotransduction, molecules, which promote the corresponding responses to mechanical load, are potential targets. Via the inhibition or augmentation of targets, therapies mimicking the enhanced mechanotransduction may increase osteogenesis to improve lost bone mass in osteoporosis patients or astronauts.

## Data Availability

This article has no additional data.

## References

[1] Sarafrazi N., Wambogo E.A., Shepherd J.A. (2021). Osteoporosis or Low Bone Mass in Older Adults: United States, 2017–2018.

[2] Kerschan-Schindl K. (2016). Prevention and Rehabilitation of Osteoporosis. Wien. Med. Wochenschr..

[3] Aspray T.J., Hill T.R. (2019). Osteoporosis and the Ageing Skeleton. Subcell. Biochem..

[4] Pietschmann P., Kerschan-Schindl K. (2004). Osteoporosis: Gender-Specific Aspects. Wien. Med. Wochenschr..

[5] Ettinger B. (1988). Prevention of Osteoporosis: Treatment of Estradiol Deficiency. Obstet. Gynecol..

[6] Rolvien T., Amling M. (2022). Disuse Osteoporosis: Clinical and Mechanistic Insights. Calcif. Tissue Int..

[7] Tilton F.E., Degioanni J.J., Schneider V.S. (1980). Long-Term Follow-up of Skylab Bone Demineralization. Aviat. Space Environ. Med..

[8] Whedon G.D., Lutwak L., Rambaut P., Whittle M., Leach C., Reid J., Smith M. (1976). Effect of Weightlessness on Mineral Metabolism; Metabolic Studies on Skylab Orbital Space Flights. Calcif. Tissue Res..

[9] Rambaut P.C., Johnston R.S. (1979). Prolonged Weightlessness and Calcium Loss in Man. Acta Astronaut..

[10] Collet P., Uebelhart D., Vico L., Moro L., Hartmann D., Roth M., Alexandre C. (1997). Effects of 1- and 6-Month Spaceflight on Bone Mass and Biochemistry in Two Humans. Bone.

[11] Sibonga J.D., Evans H.J., Sung H.G., Spector E.R., Lang T.F., Oganov V.S., Bakulin A.V., Shackelford L.C., LeBlanc A.D. (2007). Recovery of Spaceflight-Induced Bone Loss: Bone Mineral Density after Long-Duration Missions as Fitted with an Exponential Function. Bone.

[12] Vico L., Chappard D., Palle S., Bakulin A.V., Novikov V.E., Alexandre C. (1988). Trabecular Bone Remodeling after Seven Days of Weightlessness Exposure (BIOCOSMOS 1667). Am. J. Physiol..

[13] Iwamoto J., Takeda T., Sato Y. (2005). Interventions to Prevent Bone Loss in Astronauts during Space Flight. Keio J. Med..

[14] Huang C., Ogawa R. (2010). Mechanotransduction in Bone Repair and Regeneration. FASEB J..

[15] Setiawati R., Rahardjo P., Yang H. (2019). Bone Development and Growth. Osteogenesis and Bone Regeneration.

[16] Matsushita Y., Ono W., Ono N. (2020). Skeletal Stem Cells for Bone Development and Repair: Diversity Matters. Curr. Osteoporos. Rep..

[17] Solidum J.G.N., Jeong Y., Heralde F., Park D. (2023). Differential Regulation of Skeletal Stem/Progenitor Cells in Distinct Skeletal Compartments. Front. Physiol..

[18] Choi J.U.A., Kijas A.W., Lauko J., Rowan A.E. (2021). The Mechanosensory Role of Osteocytes and Implications for Bone Health and Disease States. Front. Cell Dev. Biol..

[19] Nagayama K., Kodama F., Wataya N., Sato A., Matsumoto T. (2022). Changes in the Intra- and Extra-Mechanical Environment of the Nucleus in Saos-2 Osteoblastic Cells during Bone Differentiation Process: Nuclear Shrinkage and Stiffening in Cell Differentiation. J. Mech. Behav. Biomed. Mater..

[20] Bensreti H., Alhamad D.W., Gonzalez A.M., Pizarro-Mondesir M., Bollag W.B., Isales C.M., McGee-Lawrence M.E. (2022). Update on the Role of Glucocorticoid Signaling in Osteoblasts and Bone Marrow Adipocytes During Aging. Curr. Osteoporos. Rep..

[21] Long F. (2011). Building Strong Bones: Molecular Regulation of the Osteoblast Lineage. Nat. Rev. Mol. Cell Biol..

[22] Jeong Y., Park D. (2020). Targeting Periosteal SSCs for Aged Bone Defects. Aging.

[23] Breeland G., Sinkler M.A., Menezes R.G. (2022). Embryology, Bone Ossification. StatPearls.

[24] Amarasekara D.S., Kim S., Rho J. (2021). Regulation of Osteoblast Differentiation by Cytokine Networks. Int. J. Mol. Sci..

[25] Collins F.L., Rios-Arce N.D., Schepper J.D., Parameswaran N., McCabe L.R. (2017). The Potential of Probiotics as a Therapy for Osteoporosis. Microbiol. Spectr..

[26] Bilgiç E., Boyacıoğlu Ö., Gizer M., Korkusuz P., Korkusuz F., Angin S., Şimşek I.E. (2020). Chapter 6—Architecture of Bone Tissue and Its Adaptation to Pathological Conditions. Comparative Kinesiology of the Human Body.

[27] Provot S., Schipani E., Wu J., Kronenberg H., Dempster D.W., Cauley J.A., Bouxsein M.L., Cosman F. (2021). Chapter 3—Development of the Skeleton. Marcus and Feldman’s Osteoporosis.

[28] Granéli C., Thorfve A., Ruetschi U., Brisby H., Thomsen P., Lindahl A., Karlsson C. (2014). Novel Markers of Osteogenic and Adipogenic Differentiation of Human Bone Marrow Stromal Cells Identified Using a Quantitative Proteomics Approach. Stem Cell Res..

[29] Murshed M. (2018). Mechanism of Bone Mineralization. Cold Spring Harb. Perspect. Med..

[30] Sharma U., Pal D., Prasad R. (2014). Alkaline Phosphatase: An Overview. Indian. J. Clin. Biochem..

[31] Marom R., Shur I., Solomon R., Benayahu D. (2005). Characterization of Adhesion and Differentiation Markers of Osteogenic Marrow Stromal Cells. J. Cell. Physiol..

[32] Gordon J.A.R., Tye C.E., Sampaio A.V., Underhill T.M., Hunter G.K., Goldberg H.A. (2007). Bone Sialoprotein Expression Enhances Osteoblast Differentiation and Matrix Mineralization In Vitro. Bone.

[33] Manolagas S.C. (2020). Osteocalcin Promotes Bone Mineralization but Is Not a Hormone. PLoS Genet..

[34] Holm E., Gleberzon J.S., Liao Y., Sørensen E.S., Beier F., Hunter G.K., Goldberg H.A. (2014). Osteopontin Mediates Mineralization and Not Osteogenic Cell Development in Vitro. Biochem. J..

[35] Si J., Wang C., Zhang D., Wang B., Hou W., Zhou Y. (2020). Osteopontin in Bone Metabolism and Bone Diseases. Med. Sci. Monit..

[36] Salhotra A., Shah H.N., Levi B., Longaker M.T. (2020). Mechanisms of Bone Development and Repair. Nat. Rev. Mol. Cell Biol..

[37] St John H.C., Bishop K.A., Meyer M.B., Benkusky N.A., Leng N., Kendziorski C., Bonewald L.F., Pike J.W. (2014). The Osteoblast to Osteocyte Transition: Epigenetic Changes and Response to the Vitamin D3 Hormone. Mol. Endocrinol..

[38] Brown J.L., Kumbar S.G., Laurencin C.T., Ratner B.D., Hoffman A.S., Schoen F.J., Lemons J.E. (2013). Chapter II.6.7—Bone Tissue Engineering. Biomaterials Science.

[39] Khotib J., Marhaeny H.D., Miatmoko A., Budiatin A.S., Ardianto C., Rahmadi M., Pratama Y.A., Tahir M. (2022). Differentiation of Osteoblasts: The Links between Essential Transcription Factors. J. Biomol. Struct. Dyn..

[40] Lin F.T., Lane M.D. (1994). CCAAT/Enhancer Binding Protein Alpha Is Sufficient to Initiate the 3T3-L1 Adipocyte Differentiation Program. Proc. Natl. Acad. Sci. USA.

[41] Lefebvre V., Dvir-Ginzberg M. (2017). SOX9 and the Many Facets of Its Regulation in the Chondrocyte Lineage. Connect. Tissue Res..

[42] Arboleya L., Castañeda S. (2013). Osteoimmunology: The Study of the Relationship between the Immune System and Bone Tissue. Reumatol. Clin..

[43] Maruyama Z., Yoshida C.A., Furuichi T., Amizuka N., Ito M., Fukuyama R., Miyazaki T., Kitaura H., Nakamura K., Fujita T. (2007). Runx2 Determines Bone Maturity and Turnover Rate in Postnatal Bone Development and Is Involved in Bone Loss in Estrogen Deficiency. Dev. Dyn..

[44] Qin X., Jiang Q., Miyazaki T., Komori T. (2019). Runx2 Regulates Cranial Suture Closure by Inducing Hedgehog, Fgf, Wnt and Pthlh Signaling Pathway Gene Expressions in Suture Mesenchymal Cells. Hum. Mol. Genet..

[45] Liu Q., Li M., Wang S., Xiao Z., Xiong Y., Wang G. (2020). Recent Advances of Osterix Transcription Factor in Osteoblast Differentiation and Bone Formation. Front. Cell Dev. Biol..

[46] Komori T. (2006). Regulation of Osteoblast Differentiation by Transcription Factors. J. Cell. Biochem..

[47] Zhang Y., Lin T., Lian N., Tao H., Li C., Li L., Yang X. (2019). Hop2 Interacts with ATF4 to Promote Osteoblast Differentiation. J. Bone Miner. Res..

[48] Yang X., Karsenty G. (2004). ATF4, the Osteoblast Accumulation of Which Is Determined Post-Translationally, Can Induce Osteoblast-Specific Gene Expression in Non-Osteoblastic Cells. J. Biol. Chem..

[49] Yu S., Zhu K., Lai Y., Zhao Z., Fan J., Im H.-J., Chen D., Xiao G. (2013). ATF4 Promotes β-Catenin Expression and Osteoblastic Differentiation of Bone Marrow Mesenchymal Stem Cells. Int. J. Biol. Sci..

[50] Ichida F., Nishimura R., Hata K., Matsubara T., Ikeda F., Hisada K., Yatani H., Cao X., Komori T., Yamaguchi A. (2004). Reciprocal Roles of Msx2 in Regulation of Osteoblast and Adipocyte Differentiation. J. Biol. Chem..

[51] Jochum W., David J.P., Elliott C., Wutz A., Plenk H., Matsuo K., Wagner E.F. (2000). Increased Bone Formation and Osteosclerosis in Mice Overexpressing the Transcription Factor Fra-1. Nat. Med..

[52] Kenner L., Hoebertz A., Beil F.T., Keon N., Karreth F., Eferl R., Scheuch H., Szremska A., Amling M., Schorpp-Kistner M. (2004). Mice Lacking JunB Are Osteopenic Due to Cell-Autonomous Osteoblast and Osteoclast Defects. J. Cell Biol..

[53] Nishikawa K., Nakashima T., Takeda S., Isogai M., Hamada M., Kimura A., Kodama T., Yamaguchi A., Owen M.J., Takahashi S. (2010). Maf Promotes Osteoblast Differentiation in Mice by Mediating the Age-Related Switch in Mesenchymal Cell Differentiation. J. Clin. Investig..

[54] Li H., Liu P., Xu S., Li Y., Dekker J.D., Li B., Fan Y., Zhang Z., Hong Y., Yang G. (2017). FOXP1 Controls Mesenchymal Stem Cell Commitment and Senescence during Skeletal Aging. J. Clin. Investig..

[55] Zhang M., Ma T., Hu B., Xiang W. (2022). FOXP1 Promotes Osteoblast Differentiation via Regulation of TGF-β/ALK-5 Pathway. ScienceAsia.

[56] Komori T. (2010). Regulation of Osteoblast Differentiation by Runx2. Adv. Exp. Med. Biol..

[57] Vater C., Kasten P., Stiehler M. (2011). Culture Media for the Differentiation of Mesenchymal Stromal Cells. Acta Biomater..

[58] Hanna H., Mir L.M., Andre F.M. (2018). In Vitro Osteoblastic Differentiation of Mesenchymal Stem Cells Generates Cell Layers with Distinct Properties. Stem Cell Res. Ther..

[59] Huang W., Yang S., Shao J., Li Y.-P. (2007). Signaling and Transcriptional Regulation in Osteoblast Commitment and Differentiation. Front. Biosci..

[60] Zayzafoon M., Gathings W.E., McDonald J.M. (2004). Modeled Microgravity Inhibits Osteogenic Differentiation of Human Mesenchymal Stem Cells and Increases Adipogenesis. Endocrinology.

[61] Ontiveros C., McCabe L.R. (2003). Simulated Microgravity Suppresses Osteoblast Phenotype, Runx2 Levels and AP-1 Transactivation. J. Cell. Biochem..

[62] Hu L., Li J., Qian A., Wang F., Shang P. (2015). Mineralization Initiation of MC3T3-E1 Preosteoblast Is Suppressed under Simulated Microgravity Condition. Cell Biol. Int..

[63] Wang L. (2018). Solute Transport in the Bone Lacunar-Canalicular System (LCS). Curr. Osteoporos. Rep..

[64] You L.-D., Weinbaum S., Cowin S.C., Schaffler M.B. (2004). Ultrastructure of the Osteocyte Process and Its Pericellular Matrix. Anat. Rec. Part A Discov. Mol. Cell Evol. Biol..

[65] Wang H., Du T., Li R., Main R.P., Yang H. (2022). Interactive Effects of Various Loading Parameters on the Fluid Dynamics within the Lacunar-Canalicular System for a Single Osteocyte. Bone.

[66] Rodionova N.V., Oganov V.S., Zolotova N.V. (2002). Ultrastructural Changes in Osteocytes in Microgravity Conditions. Adv. Space Res..

[67] Blaber E.A., Dvorochkin N., Lee C., Alwood J.S., Yousuf R., Pianetta P., Globus R.K., Burns B.P., Almeida E.A.C. (2013). Microgravity Induces Pelvic Bone Loss through Osteoclastic Activity, Osteocytic Osteolysis, and Osteoblastic Cell Cycle Inhibition by CDKN1a/P21. PLoS ONE.

[68] Matsuzaka T., Matsugaki A., Nakano T. (2021). Control of Osteoblast Arrangement by Osteocyte Mechanoresponse through Prostaglandin E2 Signaling under Oscillatory Fluid Flow Stimuli. Biomaterials.

[69] Tan S.D., Bakker A.D., Semeins C.M., Kuijpers-Jagtman A.M., Klein-Nulend J. (2008). Inhibition of Osteocyte Apoptosis by Fluid Flow Is Mediated by Nitric Oxide. Biochem. Biophys. Res. Commun..

[70] Sheng M.H.C., Lau K.H.W., Baylink D.J. (2014). Role of Osteocyte-Derived Insulin-Like Growth Factor I in Developmental Growth, Modeling, Remodeling, and Regeneration of the Bone. J. Bone Metab..

[71] Goldring S.R. (2015). The Osteocyte: Key Player in Regulating Bone Turnover. RMD Open.

[72] Watanabe-Takano H., Ochi H., Chiba A., Matsuo A., Kanai Y., Fukuhara S., Ito N., Sako K., Miyazaki T., Tainaka K. (2021). Mechanical Load Regulates Bone Growth via Periosteal Osteocrin. Cell Rep..

[73] Duncan R.L., Turner C.H. (1995). Mechanotransduction and the Functional Response of Bone to Mechanical Strain. Calcif. Tissue Int..

[74] Campbell I.D., Humphries M.J. (2011). Integrin Structure, Activation, and Interactions. Cold Spring Harb. Perspect. Biol..

[75] Gronthos S., Simmons P.J., Graves S.E., Robey P.G. (2001). Integrin-Mediated Interactions between Human Bone Marrow Stromal Precursor Cells and the Extracellular Matrix. Bone.

[76] Clover J., Dodds R.A., Gowen M. (1992). Integrin Subunit Expression by Human Osteoblasts and Osteoclasts in Situ and in Culture. J. Cell Sci..

[77] Qin L., Liu W., Cao H., Xiao G. (2020). Molecular Mechanosensors in Osteocytes. Bone Res..

[78] Geoghegan I.P., Hoey D.A., McNamara L.M. (2019). Estrogen Deficiency Impairs Integrin Avβ3-Mediated Mechanosensation by Osteocytes and Alters Osteoclastogenic Paracrine Signalling. Sci. Rep..

[79] Theocharis A.D., Skandalis S.S., Gialeli C., Karamanos N.K. (2016). Extracellular Matrix Structure. Adv. Drug Deliv. Rev..

[80] Saito M., Marumo K. (2015). Effects of Collagen Crosslinking on Bone Material Properties in Health and Disease. Calcif. Tissue Int..

[81] Kirby D.J., Young M.F. (2018). Isolation, Production, and Analysis of Small Leucine-Rich Proteoglycans in Bone. Methods Cell Biol..

[82] Marinovich R., Soenjaya Y., Wallace G.Q., Zuskov A., Dunkman A., Foster B.L., Ao M., Bartman K., Lam V., Rizkalla A. (2016). The Role of Bone Sialoprotein in the Tendon–Bone Insertion. Matrix Biol..

[83] Sun Z., Guo S.S., Fässler R. (2016). Integrin-Mediated Mechanotransduction. J. Cell Biol..

[84] Cheah M., Andrews M.R. (2018). Integrin Activation: Implications for Axon Regeneration. Cells.

[85] Chen W., Lou J., Evans E.A., Zhu C. (2012). Observing Force-Regulated Conformational Changes and Ligand Dissociation from a Single Integrin on Cells. J. Cell Biol..

[86] Kong F., Li Z., Parks W.M., Dumbauld D.W., García A.J., Mould A.P., Humphries M.J., Zhu C. (2013). Cyclic Mechanical Reinforcement of Integrin-Ligand Interactions. Mol. Cell.

[87] Oria R., Wiegand T., Escribano J., Elosegui-Artola A., Uriarte J.J., Moreno-Pulido C., Platzman I., Delcanale P., Albertazzi L., Navajas D. (2017). Force Loading Explains Spatial Sensing of Ligands by Cells. Nature.

[88] Strohmeyer N., Bharadwaj M., Costell M., Fässler R., Müller D.J. (2017). Fibronectin-Bound A5β1 Integrins Sense Load and Signal to Reinforce Adhesion in Less than a Second. Nat. Mater..

[89] Moursi A.M., Globus R.K., Damsky C.H. (1997). Interactions between Integrin Receptors and Fibronectin Are Required for Calvarial Osteoblast Differentiation in Vitro. J. Cell Sci..

[90] To W.S., Midwood K.S. (2011). Plasma and Cellular Fibronectin: Distinct and Independent Functions during Tissue Repair. Fibrogenes. Tissue Repair..

[91] Meyers V.E., Zayzafoon M., Gonda S.R., Gathings W.E., McDonald J.M. (2004). Modeled Microgravity Disrupts Collagen I/Integrin Signaling during Osteoblastic Differentiation of Human Mesenchymal Stem Cells. J. Cell. Biochem..

[92] Martinac B., Poole K. (2018). Mechanically Activated Ion Channels. Int. J. Biochem. Cell Biol..

[93] Ranade S.S., Syeda R., Patapoutian A. (2015). Mechanically Activated Ion Channels. Neuron.

[94] Zhu K., Prince R.L. (2012). Calcium and Bone. Clin. Biochem..

[95] Nakamura S., Matsumoto T., Sasaki J.-I., Egusa H., Lee K.Y., Nakano T., Sohmura T., Nakahira A. (2010). Effect of Calcium Ion Concentrations on Osteogenic Differentiation and Hematopoietic Stem Cell Niche-Related Protein Expression in Osteoblasts. Tissue Eng. Part A.

[96] Blair H.C., Schlesinger P.H., Huang C.L.H., Zaidi M. (2007). Calcium Signalling and Calcium Transport in Bone Disease. Subcell. Biochem..

[97] Coste B., Mathur J., Schmidt M., Earley T.J., Ranade S., Petrus M.J., Dubin A.E., Patapoutian A. (2010). Piezo1 and Piezo2 Are Essential Components of Distinct Mechanically Activated Cation Channels. Science.

[98] Wang L., Zhou H., Zhang M., Liu W., Deng T., Zhao Q., Li Y., Lei J., Li X., Xiao B. (2019). Structure and Mechanogating of the Mammalian Tactile Channel PIEZO2. Nature.

[99] Zhao Q., Zhou H., Chi S., Wang Y., Wang J., Geng J., Wu K., Liu W., Zhang T., Dong M.-Q. (2018). Structure and Mechanogating Mechanism of the Piezo1 Channel. Nature.

[100] Jiang Y., Yang X., Jiang J., Xiao B. (2021). Structural Designs and Mechanogating Mechanisms of the Mechanosensitive Piezo Channels. Trends Biochem. Sci..

[101] Zhao Q., Zhou H., Li X., Xiao B. (2019). The Mechanosensitive Piezo1 Channel: A Three-bladed Propeller-like Structure and a Lever-like Mechanogating Mechanism. FEBS J..

[102] Sugimoto A., Miyazaki A., Kawarabayashi K., Shono M., Akazawa Y., Hasegawa T., Ueda-Yamaguchi K., Kitamura T., Yoshizaki K., Fukumoto S. (2017). Piezo Type Mechanosensitive Ion Channel Component 1 Functions as a Regulator of the Cell Fate Determination of Mesenchymal Stem Cells. Sci. Rep..

[103] Sun W., Chi S., Li Y., Ling S., Tan Y., Xu Y., Jiang F., Li J., Liu C., Zhong G. (2019). The Mechanosensitive Piezo1 Channel Is Required for Bone Formation. eLife.

[104] Song J., Liu L., Lv L., Hu S., Tariq A., Wang W., Dang X. (2020). Fluid Shear Stress Induces Runx-2 Expression via Upregulation of PIEZO1 in MC3T3-E1 Cells. Cell Biol. Int..

[105] Zhang G., Li X., Wu L., Qin Y.-X. (2021). Piezo1 Channel Activation in Response to Mechanobiological Acoustic Radiation Force in Osteoblastic Cells. Bone Res..

[106] Syeda R., Florendo M.N., Cox C.D., Kefauver J.M., Santos J.S., Martinac B., Patapoutian A. (2016). Piezo1 Channels Are Inherently Mechanosensitive. Cell Rep..

[107] Geng J., Liu W., Zhou H., Zhang T., Wang L., Zhang M., Li Y., Shen B., Li X., Xiao B. (2020). A Plug-and-Latch Mechanism for Gating the Mechanosensitive Piezo Channel. Neuron.

[108] Geng J., Zhao Q., Zhang T., Xiao B. (2017). In Touch With the Mechanosensitive Piezo Channels: Structure, Ion Permeation, and Mechanotransduction. Curr. Top. Membr..

[109] Zhao Q., Wu K., Geng J., Chi S., Wang Y., Zhi P., Zhang M., Xiao B. (2016). Ion Permeation and Mechanotransduction Mechanisms of Mechanosensitive Piezo Channels. Neuron.

[110] Chinipardaz Z., Liu M., Graves D.T., Yang S. (2022). Role of Primary Cilia in Bone and Cartilage. J. Dent. Res..

[111] Malone A.M.D., Anderson C.T., Tummala P., Kwon R.Y., Johnston T.R., Stearns T., Jacobs C.R. (2007). Primary Cilia Mediate Mechanosensing in Bone Cells by a Calcium-Independent Mechanism. Proc. Natl. Acad. Sci. USA.

[112] Xiao Z., Zhang S., Mahlios J., Zhou G., Magenheimer B.S., Guo D., Dallas S.L., Maser R., Calvet J.P., Bonewald L. (2006). Cilia-like Structures and Polycystin-1 in Osteoblasts/Osteocytes and Associated Abnormalities in Skeletogenesis and Runx2 Expression. J. Biol. Chem..

[113] Tummala P., Arnsdorf E.J., Jacobs C.R. (2010). The Role of Primary Cilia in Mesenchymal Stem Cell Differentiation: A Pivotal Switch in Guiding Lineage Commitment. Cell. Mol. Bioeng..

[114] Schwartz E.A., Leonard M.L., Bizios R., Bowser S.S. (1997). Analysis and Modeling of the Primary Cilium Bending Response to Fluid Shear. Am. J. Physiol..

[115] Hoey D.A., Chen J.C., Jacobs C.R. (2012). The Primary Cilium as a Novel Extracellular Sensor in Bone. Front. Endocrinol..

[116] You J., Reilly G.C., Zhen X., Yellowley C.E., Chen Q., Donahue H.J., Jacobs C.R. (2001). Osteopontin Gene Regulation by Oscillatory Fluid Flow via Intracellular Calcium Mobilization and Activation of Mitogen-Activated Protein Kinase in MC3T3-E1 Osteoblasts. J. Biol. Chem..

[117] Deng Z., Paknejad N., Maksaev G., Sala-Rabanal M., Nichols C.G., Hite R.K., Yuan P. (2018). Cryo-EM and X-Ray Structures of TRPV4 Reveal Insight into Ion Permeation and Gating Mechanisms. Nat. Struct. Mol. Biol..

[118] Everaerts W., Nilius B., Owsianik G. (2010). The Vanilloid Transient Receptor Potential Channel TRPV4: From Structure to Disease. Prog. Biophys. Mol. Biol..

[119] Das R., Goswami C. (2019). TRPV4 Expresses in Bone Cell Lineages and TRPV4-R616Q Mutant Causing Brachyolmia in Human Reveals “Loss-of-Interaction” with Cholesterol. Biochem. Biophys. Res. Commun..

[120] Son A., Kang N., Kang J.Y., Kim K.W., Yang Y.-M., Shin D.M. (2018). TRPM3/TRPV4 Regulates Ca^2+^-Mediated RANKL/NFATc1 Expression in Osteoblasts. J. Mol. Endocrinol..

[121] Williams K.M., Leser J.M., Gould N.R., Joca H.C., Lyons J.S., Khairallah R.J., Ward C.W., Stains J.P. (2020). TRPV4 Calcium Influx Controls Sclerostin Protein Loss Independent of Purinergic Calcium Oscillations. Bone.

[122] Masuyama R., Vriens J., Voets T., Karashima Y., Owsianik G., Vennekens R., Lieben L., Torrekens S., Moermans K., Vanden Bosch A. (2008). TRPV4-Mediated Calcium Influx Regulates Terminal Differentiation of Osteoclasts. Cell Metab..

[123] Liu N., Lu W., Dai X., Qu X., Zhu C. (2022). The Role of TRPV Channels in Osteoporosis. Mol. Biol. Rep..

[124] Nishimura G., Lausch E., Savarirayan R., Shiba M., Spranger J., Zabel B., Ikegawa S., Superti-Furga A., Unger S. (2012). TRPV4-Associated Skeletal Dysplasias. Am. J. Med. Genet. Part C Semin. Med. Genet..

[125] Suzuki T., Notomi T., Miyajima D., Mizoguchi F., Hayata T., Nakamoto T., Hanyu R., Kamolratanakul P., Mizuno A., Suzuki M. (2013). Osteoblastic Differentiation Enhances Expression of TRPV4 That Is Required for Calcium Oscillation Induced by Mechanical Force. Bone.

[126] Warita K., Aoki R., Kitamura N., Shibuya I., Hosaka Y.Z. (2019). The Precursor Osteoblast-like Cell, MC3T3-E1 Cell Line, Enhances Sodium-Calcium Exchanger 1 (Ncx1) Gene Expression by Stretch Stimuli Prior to Osteoblast Differentiation. J. Vet. Med. Sci..

[127] Corrigan M.A., Johnson G.P., Stavenschi E., Riffault M., Labour M.-N., Hoey D.A. (2018). TRPV4-Mediates Oscillatory Fluid Shear Mechanotransduction in Mesenchymal Stem Cells in Part via the Primary Cilium. Sci. Rep..

[128] Mollazadeh S., Fazly Bazzaz B.S., Kerachian M.A. (2015). Role of Apoptosis in Pathogenesis and Treatment of Bone-Related Diseases. J. Orthop. Surg. Res..

[129] Batra N., Kar R., Jiang J.X. (2012). Gap Junctions and Hemichannels in Signal Transmission, Function and Development of Bone. Biochim. Biophys. Acta.

[130] Mikami Y., Yamamoto K., Akiyama Y., Kobayashi M., Watanabe E., Watanabe N., Asano M., Shimizu N., Komiyama K. (2015). Osteogenic Gene Transcription Is Regulated via Gap Junction-Mediated Cell–Cell Communication. Stem Cells Dev..

[131] Buo A.M., Stains J.P. (2014). Gap Junctional Regulation of Signal Transduction in Bone Cells. FEBS Lett..

[132] Jiang J.X., Siller-Jackson A.J., Burra S. (2007). Roles of Gap Junctions and Hemichannels in Bone Cell Functions and in Signal Transmission of Mechanical Stress. Front. Biosci..

[133] Plotkin L.I. (2011). Connexin 43 and bone: Not just a gap junction protein. Actual. Osteol..

[134] Loiselle A.E., Jiang J.X., Donahue H.J. (2013). Gap Junction and Hemichannel Functions in Osteocytes. Bone.

[135] Civitelli R. (2008). Cell-Cell Communication in the Osteoblast/Osteocyte Lineage. Arch. Biochem. Biophys..

[136] Robinson J.A., Chatterjee-Kishore M., Yaworsky P.J., Cullen D.M., Zhao W., Li C., Kharode Y., Sauter L., Babij P., Brown E.L. (2006). Wnt/Beta-Catenin Signaling Is a Normal Physiological Response to Mechanical Loading in Bone. J. Biol. Chem..

[137] Mbalaviele G., Shin C.S., Civitelli R. (2006). Cell-Cell Adhesion and Signaling through Cadherins: Connecting Bone Cells in Their Microenvironment. J. Bone Miner. Res..

[138] Marie P.J., Haÿ E., Modrowski D., Revollo L., Mbalaviele G., Civitelli R. (2014). Cadherin-Mediated Cell-Cell Adhesion and Signaling in the Skeleton. Calcif. Tissue Int..

[139] Marie P.J. (2002). Role of N-Cadherin in Bone Formation. J. Cell. Physiol..

[140] Castro C.H.M., Shin C.S., Stains J.P., Cheng S.-L., Sheikh S., Mbalaviele G., Szejnfeld V.L., Civitelli R. (2004). Targeted Expression of a Dominant-Negative N-Cadherin in Vivo Delays Peak Bone Mass and Increases Adipogenesis. J. Cell Sci..

[141] Arnsdorf E.J., Tummala P., Jacobs C.R. (2009). Non-Canonical Wnt Signaling and N-Cadherin Related β-Catenin Signaling Play a Role in Mechanically Induced Osteogenic Cell Fate. PLoS ONE.

[142] Stewart S., Darwood A., Masouros S., Higgins C., Ramasamy A. (2020). Mechanotransduction in Osteogenesis. Bone Joint Res..

[143] Ingber D.E. (1993). Cellular Tensegrity: Defining New Rules of Biological Design That Govern the Cytoskeleton. J. Cell Sci..

[144] Nonaka S., Naoki H., Ishii S. (2011). A Multiphysical Model of Cell Migration Integrating Reaction-Diffusion, Membrane and Cytoskeleton. Neural Netw..

[145] Porshneva K., Montagnac G. (2022). Mechanotransduction Mediated by Microtubules. Nat. Mater..

[146] Sanghvi-Shah R., Weber G.F. (2017). Intermediate Filaments at the Junction of Mechanotransduction, Migration, and Development. Front. Cell Dev. Biol..

[147] Lu S., Wang Y., Engler A.J., Kumar S. (2014). Chapter Two—Single-Cell Imaging of Mechanotransduction in Endothelial Cells. Progress in Molecular Biology and Translational Science.

[148] Critchley D.R. (2004). Cytoskeletal Proteins Talin and Vinculin in Integrin-Mediated Adhesion. Biochem. Soc. Trans..

[149] Critchley D.R. (2005). Genetic, Biochemical and Structural Approaches to Talin Function. Biochem. Soc. Trans..

[150] Urciuoli E., Peruzzi B. (2020). Involvement of the FAK Network in Pathologies Related to Altered Mechanotransduction. Int. J. Mol. Sci..

[151] Moreno-Layseca P., Icha J., Hamidi H., Ivaska J. (2019). Integrin Trafficking in Cells and Tissues. Nat. Cell Biol..

[152] Humphries J.D., Wang P., Streuli C., Geiger B., Humphries M.J., Ballestrem C. (2007). Vinculin Controls Focal Adhesion Formation by Direct Interactions with Talin and Actin. J. Cell Biol..

[153] Freitas F., Jeschke M., Majstorovic I., Mueller D.R., Schindler P., Voshol H., Van Oostrum J., Susa M. (2002). Fluoroaluminate Stimulates Phosphorylation of P130 Cas and Fak and Increases Attachment and Spreading of Preosteoblastic MC3T3-E1 Cells. Bone.

[154] Zhao X., Guan J.-L. (2011). Focal Adhesion Kinase and Its Signaling Pathways in Cell Migration and Angiogenesis. Adv. Drug Deliv. Rev..

[155] Miyazaki T., Zhao Z., Ichihara Y., Yoshino D., Imamura T., Sawada K., Hayano S., Kamioka H., Mori S., Hirata H. (2019). Mechanical Regulation of Bone Homeostasis through p130Cas-Mediated Alleviation of NF-κB Activity. Sci. Adv..

[156] Bertrand A.A., Malapati S.H., Yamaguchi D.T., Lee J.C. (2020). The Intersection of Mechanotransduction and Regenerative Osteogenic Materials. Adv. Healthc. Mater..

[157] Mitra S.K., Hanson D.A., Schlaepfer D.D. (2005). Focal Adhesion Kinase: In Command and Control of Cell Motility. Nat. Rev. Mol. Cell Biol..

[158] Mitra S.K., Schlaepfer D.D. (2006). Integrin-Regulated FAK–Src Signaling in Normal and Cancer Cells. Curr. Opin. Cell Biol..

[159] Hamamura K., Swarnkar G., Tanjung N., Cho E., Li J., Na S., Yokota H. (2012). RhoA-Mediated Signaling in Mechanotransduction of Osteoblasts. Connect. Tissue Res..

[160] Zhou X., Zheng Y. (2013). Cell Type-Specific Signaling Function of RhoA GTPase: Lessons from Mouse Gene Targeting. J. Biol. Chem..

[161] Garcia-Mata R., Boulter E., Burridge K. (2011). The “Invisible Hand”: Regulation of RHO GTPases by RHOGDIs. Nat. Rev. Mol. Cell Biol..

[162] Bos J.L., Rehmann H., Wittinghofer A. (2007). GEFs and GAPs: Critical Elements in the Control of Small G Proteins. Cell.

[163] Rossman K.L., Der C.J., Sondek J. (2005). GEF Means Go: Turning on RHO GTPases with Guanine Nucleotide-Exchange Factors. Nat. Rev. Mol. Cell Biol..

[164] Narumiya S., Tanji M., Ishizaki T. (2009). Rho Signaling, ROCK and mDia1, in Transformation, Metastasis and Invasion. Cancer Metastasis Rev..

[165] Deng Z., Jia Y., Liu H., He M., Yang Y., Xiao W., Li Y. (2019). RhoA/ROCK Pathway: Implication in Osteoarthritis and Therapeutic Targets. Am. J. Transl. Res..

[166] Watanabe N., Kato T., Fujita A., Ishizaki T., Narumiya S. (1999). Cooperation between mDia1 and ROCK in Rho-Induced Actin Reorganization. Nat. Cell Biol..

[167] Amano M., Ito M., Kimura K., Fukata Y., Chihara K., Nakano T., Matsuura Y., Kaibuchi K. (1996). Phosphorylation and Activation of Myosin by Rho-Associated Kinase (Rho-Kinase). J. Biol. Chem..

[168] Kimura K., Ito M., Amano M., Chihara K., Fukata Y., Nakafuku M., Yamamori B., Feng J., Nakano T., Okawa K. (1996). Regulation of Myosin Phosphatase by Rho and Rho-Associated Kinase (Rho-Kinase). Science.

[169] Maekawa M., Ishizaki T., Boku S., Watanabe N., Fujita A., Iwamatsu A., Obinata T., Ohashi K., Mizuno K., Narumiya S. (1999). Signaling from Rho to the Actin Cytoskeleton through Protein Kinases ROCK and LIM-Kinase. Science.

[170] McBeath R., Pirone D.M., Nelson C.M., Bhadriraju K., Chen C.S. (2004). Cell Shape, Cytoskeletal Tension, and RhoA Regulate Stem Cell Lineage Commitment. Dev. Cell.

[171] Schakenraad K., Ernst J., Pomp W., Danen E.H.J., Merks R.M.H., Schmidt T., Giomi L. (2020). Mechanical Interplay between Cell Shape and Actin Cytoskeleton Organization. Soft Matter.

[172] Testa F., Palombo A., Dinicola S., D’Anselmi F., Proietti S., Pasqualato A., Masiello M.G., Coluccia P., Cucina A., Bizzarri M. (2014). Fractal Analysis of Shape Changes in Murine Osteoblasts Cultured under Simulated Microgravity. Rend. Lincei.

[173] Fletcher D.A., Mullins R.D. (2010). Cell Mechanics and the Cytoskeleton. Nature.

[174] Bradbury P., Wu H., Choi J.U., Rowan A.E., Zhang H., Poole K., Lauko J., Chou J. (2020). Modeling the Impact of Microgravity at the Cellular Level: Implications for Human Disease. Front. Cell Dev. Biol..

[175] Wu X.-T., Yang X., Tian R., Li Y.-H., Wang C.-Y., Fan Y.-B., Sun L.-W. (2022). Cells Respond to Space Microgravity through Cytoskeleton Reorganization. FASEB J..

[176] Janmaleki M., Pachenari M., Seyedpour S.M., Shahghadami R., Sanati-Nezhad A. (2016). Impact of Simulated Microgravity on Cytoskeleton and Viscoelastic Properties of Endothelial Cell. Sci. Rep..

[177] Endo M. (2006). Calcium Ion as a Second Messenger with Special Reference to Excitation-Contraction Coupling. J. Pharmacol. Sci..

[178] Zhou T., Gao B., Fan Y., Liu Y., Feng S., Cong Q., Zhang X., Zhou Y., Yadav P.S., Lin J. (2020). Piezo1/2 Mediate Mechanotransduction Essential for Bone Formation through Concerted Activation of NFAT-YAP1-ß-Catenin. eLife.

[179] Ren R., Guo J., Chen Y., Zhang Y., Chen L., Xiong W. (2021). The Role of Ca^2+^/Calcineurin/NFAT Signalling Pathway in Osteoblastogenesis. Cell Prolif..

[180] Koga T., Matsui Y., Asagiri M., Kodama T., de Crombrugghe B., Nakashima K., Takayanagi H. (2005). NFAT and Osterix Cooperatively Regulate Bone Formation. Nat. Med..

[181] Zayzafoon M. (2006). Calcium/Calmodulin Signaling Controls Osteoblast Growth and Differentiation. J. Cell. Biochem..

[182] Kanno T., Takahashi T., Tsujisawa T., Ariyoshi W., Nishihara T. (2007). Mechanical Stress-Mediated Runx2 Activation Is Dependent on Ras/ERK1/2 MAPK Signaling in Osteoblasts. J. Cell. Biochem..

[183] Kalli M., Li R., Mills G.B., Stylianopoulos T., Zervantonakis I.K. (2022). Mechanical Stress Signaling in Pancreatic Cancer Cells Triggers P38 MAPK- and JNK-Dependent Cytoskeleton Remodeling and Promotes Cell Migration via Rac1/Cdc42/Myosin II. Mol. Cancer Res..

[184] Thouverey C., Caverzasio J. (2015). Focus on the P38 MAPK Signaling Pathway in Bone Development and Maintenance. Bonekey Rep..

[185] Rubin J., Rubin C., Jacobs C.R. (2006). Molecular Pathways Mediating Mechanical Signaling in Bone. Gene.

[186] Li Y., Ge C., Franceschi R.T. (2017). MAP Kinase-Dependent RUNX2 Phosphorylation Is Necessary for Epigenetic Modification of Chromatin During Osteoblast Differentiation. J. Cell. Physiol..

[187] Yuan X., Yang S. (2016). Primary Cilia and Intraflagellar Transport Proteins in Bone and Cartilage. J. Dent. Res..

[188] Zhang X., Schwarz E.M., Young D.A., Puzas J.E., Rosier R.N., O’Keefe R.J. (2002). Cyclooxygenase-2 Regulates Mesenchymal Cell Differentiation into the Osteoblast Lineage and Is Critically Involved in Bone Repair. J. Clin. Investig..

[189] Tuson M., He M., Anderson K.V. (2011). Protein Kinase A Acts at the Basal Body of the Primary Cilium to Prevent Gli2 Activation and Ventralization of the Mouse Neural Tube. Development.

[190] Martino F., Perestrelo A.R., Vinarský V., Pagliari S., Forte G. (2018). Cellular Mechanotransduction: From Tension to Function. Front. Physiol..

[191] Kim D.I., Birendra K.C., Roux K.J. (2015). Making the LINC: SUN and KASH Protein Interactions. Biol. Chem..

[192] Jahed Z., Domkam N., Ornowski J., Yerima G., Mofrad M.R.K. (2021). Molecular Models of LINC Complex Assembly at the Nuclear Envelope. J. Cell Sci..

[193] Kirby T.J., Lammerding J. (2018). Emerging Views of the Nucleus as a Cellular Mechanosensor. Nat. Cell Biol..

[194] Hughes-Fulford M., Lewis M.L. (1996). Effects of Microgravity on Osteoblast Growth Activation. Exp. Cell Res..

[195] Liashkovich I., Meyring A., Kramer A., Shahin V. (2011). Exceptional Structural and Mechanical Flexibility of the Nuclear Pore Complex. J. Cell. Physiol..

[196] Elosegui-Artola A., Andreu I., Beedle A.E.M., Lezamiz A., Uroz M., Kosmalska A.J., Oria R., Kechagia J.Z., Rico-Lastres P., Le Roux A.-L. (2017). Force Triggers YAP Nuclear Entry by Regulating Transport across Nuclear Pores. Cell.

[197] Vassilev A., Kaneko K.J., Shu H., Zhao Y., DePamphilis M.L. (2001). TEAD/TEF Transcription Factors Utilize the Activation Domain of YAP65, a Src/Yes-Associated Protein Localized in the Cytoplasm. Genes Dev..

[198] Kegelman C.D., Mason D.E., Dawahare J.H., Horan D.J., Vigil G.D., Howard S.S., Robling A.G., Bellido T.M., Boerckel J.D. (2018). Skeletal Cell YAP and TAZ Combinatorially Promote Bone Development. FASEB J..

[199] Pan J.-X., Xiong L., Zhao K., Zeng P., Wang B., Tang F.-L., Sun D., Guo H., Yang X., Cui S. (2018). YAP Promotes Osteogenesis and Suppresses Adipogenic Differentiation by Regulating β-Catenin Signaling. Bone Res..

[200] Byun M.R., Kim A.R., Hwang J.-H., Sung M.K., Lee Y.K., Hwang B.S., Rho J.-R., Hwang E.S., Hong J.-H. (2012). Phorbaketal A Stimulates Osteoblast Differentiation through TAZ Mediated Runx2 Activation. FEBS Lett..

[201] Xiong J., Almeida M., O’Brien C.A. (2018). The YAP/TAZ Transcriptional Co-Activators Have Opposing Effects at Different Stages of Osteoblast Differentiation. Bone.

[202] Myers R.L., Yousefi M., Lengner C.J., Klein P.S., Boffetta P., Hainaut P. (2019). Wnt Signaling in Intestinal Stem Cells and Cancer. Encyclopedia of Cancer.

[203] Bathaie S.Z., Faridi N., Nasimian A., Heidarzadeh H., Tamanoi F., Bathaie S.Z., Tamanoi F. (2015). Chapter One—How Phytochemicals Prevent Chemical Carcinogens and/or Suppress Tumor Growth?. The Enzymes.

[204] Liu C., Regimbald-Dumas Y., Zhang X., He X., Bradshaw R.A., Stahl P.D. (2016). The Wnt/β-Catenin Pathway. Encyclopedia of Cell Biology.

[205] MacDonald B.T., Tamai K., He X. (2009). Wnt/β-Catenin Signaling: Components, Mechanisms, and Diseases. Dev. Cell.

[206] Chen J., Long F. (2013). β-Catenin Promotes Bone Formation and Suppresses Bone Resorption in Postnatal Growing Mice. J. Bone Miner. Res..

[207] Case N., Rubin J. (2010). β-Catenin—A Supporting Role in the Skeleton. J. Cell. Biochem..

[208] Mao L., Wang L., Xu J., Zou J. (2023). The Role of Integrin Family in Bone Metabolism and Tumor Bone Metastasis. Cell Death Discov..

[209] Yao M., Tijore A., Cheng D., Li J.V., Hariharan A., Martinac B., Tran Van Nhieu G., Cox C.D., Sheetz M. (2022). Force- and Cell State–Dependent Recruitment of Piezo1 Drives Focal Adhesion Dynamics and Calcium Entry. Sci. Adv..

[210] Albarrán-Juárez J., Iring A., Wang S., Joseph S., Grimm M., Strilic B., Wettschureck N., Althoff T.F., Offermanns S. (2018). Piezo1 and Gq/G11 Promote Endothelial Inflammation Depending on Flow Pattern and Integrin Activation. J. Exp. Med..

[211] Hepler P.K. (2016). The Cytoskeleton and Its Regulation by Calcium and Protons. Plant Physiol..

[212] Li F., Wang W., Gu M., Gyoneva S., Zhang J., Huang S., Traynelis S.F., Cai H., Guggino S.E., Zhang X. (2011). L-Type Calcium Channel Activity in Osteoblast Cells Is Regulated by the Actin Cytoskeleton Independent of Protein Trafficking. J. Bone Miner. Metab..

[213] Gould N.R., Torre O.M., Leser J.M., Stains J.P. (2021). The Cytoskeleton and Connected Elements in Bone Cell Mechano-Transduction. Bone.

[214] Hong J.-H., Hwang E.S., McManus M.T., Amsterdam A., Tian Y., Kalmukova R., Mueller E., Benjamin T., Spiegelman B.M., Sharp P.A. (2005). TAZ, a Transcriptional Modulator of Mesenchymal Stem Cell Differentiation. Science.

[215] Zaidi S.K., Sullivan A.J., Medina R., Ito Y., van Wijnen A.J., Stein J.L., Lian J.B., Stein G.S. (2004). Tyrosine Phosphorylation Controls Runx2-Mediated Subnuclear Targeting of YAP to Repress Transcription. EMBO J..

[216] Gaur T., Lengner C.J., Hovhannisyan H., Bhat R.A., Bodine P.V.N., Komm B.S., Javed A., van Wijnen A.J., Stein J.L., Stein G.S. (2005). Canonical WNT Signaling Promotes Osteogenesis by Directly Stimulating Runx2 Gene Expression. J. Biol. Chem..

[217] Nardone G., Oliver-De La Cruz J., Vrbsky J., Martini C., Pribyl J., Skládal P., Pešl M., Caluori G., Pagliari S., Martino F. (2017). YAP Regulates Cell Mechanics by Controlling Focal Adhesion Assembly. Nat. Commun..

[218] Sebestyen J.F., Srivastava T., Alon U.S. (2012). Bisphosphonates Use in Children. Clin. Pediatr..

[219] Green J.R. (2004). Bisphosphonates: Preclinical Review. Oncologist.

[220] Sharpe M., Noble S., Spencer C.M. (2001). Alendronate: An Update of Its Use in Osteoporosis. Drugs.

[221] Waltman N., Kupzyk K.A., Flores L.E., Mack L.R., Lappe J.M., Bilek L.D. (2022). Bone-Loading Exercises versus Risedronate for the Prevention of Osteoporosis in Postmenopausal Women with Low Bone Mass: A Randomized Controlled Trial. Osteoporos. Int..

[222] Frampton J.E., Perry C.M. (2008). Ibandronate: A Review of Its Use in the Management of Postmenopausal Osteoporosis. Drugs.

[223] Dhillon S. (2016). Zoledronic Acid (Reclast^®^, Aclasta^®^): A Review in Osteoporosis. Drugs.

[224] Okada A., Matsumoto T., Ohshima H., Isomura T., Koga T., Yasui T., Kohri K., LeBlanc A., Spector E., Jones J. (2021). Bisphosphonate Use May Reduce the Risk of Urolithiasis in Astronauts on Long-Term Spaceflights. JBMR Plus.

[225] Brown J.P. (2021). Long-Term Treatment of Postmenopausal Osteoporosis. Endocrinol. Metab..

[226] Lorentzon M. (2019). Treating Osteoporosis to Prevent Fractures: Current Concepts and Future Developments. J. Intern. Med..

[227] Vargas-Franco J.W., Castaneda B., Rédiní F., Gómez D.F., Heymann D., Lézot F. (2018). Paradoxical Side Effects of Bisphosphonates on the Skeleton: What Do We Know and What Can We Do?. J. Cell. Physiol..

[228] Gendelman O., Tripto-Shkolnik L., Vered I., Lidar M. (2022). Bisphosphonates Related Ocular Side Effects: A Case Series and Review of Literature. Ocul. Immunol. Inflamm..

[229] Miller P.D., Pannacciulli N., Malouf-Sierra J., Singer A., Czerwiński E., Bone H.G., Wang C., Huang S., Chines A., Lems W. (2020). Efficacy and Safety of Denosumab vs. Bisphosphonates in Postmenopausal Women Previously Treated with Oral Bisphosphonates. Osteoporos. Int..

[230] Pittman K., Antill Y.C., Goldrick A., Goh J., de Boer R.H. (2017). Denosumab: Prevention and Management of Hypocalcemia, Osteonecrosis of the Jaw and Atypical Fractures. Asia Pac. J. Clin. Oncol..

[231] Yuan F., Peng W., Yang C., Zheng J. (2019). Teriparatide versus Bisphosphonates for Treatment of Postmenopausal Osteoporosis: A Meta-Analysis. Int. J. Surg..

[232] Sleeman A., Clements J.N. (2019). Abaloparatide: A New Pharmacological Option for Osteoporosis. Am. J. Health Syst. Pharm..

[233] Cosman F., Crittenden D.B., Adachi J.D., Binkley N., Czerwinski E., Ferrari S., Hofbauer L.C., Lau E., Lewiecki E.M., Miyauchi A. (2016). Romosozumab Treatment in Postmenopausal Women with Osteoporosis. N. Engl. J. Med..

[234] Chiba K., Okazaki N., Kurogi A., Watanabe T., Mori A., Suzuki N., Adachi K., Era M., Yokota K., Inoue T. (2022). Randomized Controlled Trial of Daily Teriparatide, Weekly High-Dose Teriparatide, or Bisphosphonate in Patients with Postmenopausal Osteoporosis: The TERABIT Study. Bone.

[235] Eriksen E.F., Robins D.A. (2004). Teriparatide: A Bone Formation Treatment for Osteoporosis. Drugs Today.

[236] Greenspan S.L., Vujevich K., Britton C., Herradura A., Gruen G., Tarkin I., Siska P., Hamlin B., Perera S. (2018). Teriparatide for Treatment of Patients with Bisphosphonate-Associated Atypical Fracture of the Femur. Osteoporos. Int..

[237] Lindsay R., Krege J.H., Marin F., Jin L., Stepan J.J. (2016). Teriparatide for Osteoporosis: Importance of the Full Course. Osteoporos. Int..

[238] Shane E., Shiau S., Recker R.R., Lappe J.M., Agarwal S., Kamanda-Kosseh M., Bucovsky M., Stubby J., Cohen A. (2022). Denosumab After Teriparatide in Premenopausal Women With Idiopathic Osteoporosis. J. Clin. Endocrinol. Metab..

[239] Rengel A., Tran V., Toh L.S. (2023). Denosumab as a Pharmacological Countermeasure against Osteopenia in Long Duration Spaceflight. Aerosp. Med. Hum. Perform..

[240] Li L., Zhu Z., Huang C., Chen W. (2008). Ultrasound: A Potential Technique to Improve Osseointegration of Dental Implants. Med. Hypotheses.

[241] Victoria G., Petrisor B., Drew B., Dick D. (2009). Bone Stimulation for Fracture Healing: What’s All the Fuss?. Indian. J. Orthop..

[242] Higgins A., Glover M., Yang Y., Bayliss S., Meads C., Lord J. (2014). EXOGEN Ultrasound Bone Healing System for Long Bone Fractures with Non-Union or Delayed Healing: A NICE Medical Technology Guidance. Appl. Health Econ. Health Policy.

[243] Yang W., Huo X., Song T. (2008). Effects of Extremely Low-Frequency-Pulsed Electromagnetic Field on Different-Derived Osteoblast-like Cells. Electromagn. Biol. Med..

[244] Xue Y., Hu S., Chen C., He J., Sun J., Jin Y., Zhang Y., Zhu G., Shi Q., Rui Y. (2022). Myokine Irisin Promotes Osteogenesis by Activating BMP/SMAD Signaling via αV Integrin and Regulates Bone Mass in Mice. Int. J. Biol. Sci..

[245] Liang C., Guo B., Wu H., Shao N., Li D., Liu J., Dang L., Wang C., Li H., Li S. (2015). Aptamer-Functionalized Lipid Nanoparticles Targeting Osteoblasts as a Novel RNA Interference–Based Bone Anabolic Strategy. Nat. Med..

[246] Stapleton M., Sawamoto K., Alméciga-Díaz C.J., Mackenzie W.G., Mason R.W., Orii T., Tomatsu S. (2017). Development of Bone Targeting Drugs. Int. J. Mol. Sci..

[247] Cui Y., Guo Y., Kong L., Shi J., Liu P., Li R., Geng Y., Gao W., Zhang Z., Fu D. (2022). A Bone-Targeted Engineered Exosome Platform Delivering siRNA to Treat Osteoporosis. Bioact. Mater..

[248] Gao Y., Xin H., Cai B., Wang L., Lv Q., Hou Y., Liu F., Dai T., Kong L. (2022). RNA Interference-Based Osteoanabolic Therapy for Osteoporosis by a Bone-Formation Surface Targeting Delivery System. Biochem. Biophys. Res. Commun..

[249] Guo J., Wang F., Hu Y., Luo Y., Wei Y., Xu K., Zhang H., Liu H., Bo L., Lv S. (2023). Exosome-Based Bone-Targeting Drug Delivery Alleviates Impaired Osteoblastic Bone Formation and Bone Loss in Inflammatory Bowel Diseases. Cell Rep. Med..

[250] Zheng L., Zhuang Z., Li Y., Shi T., Fu K., Yan W., Zhang L., Wang P., Li L., Jiang Q. (2021). Bone Targeting Antioxidative Nano-Iron Oxide for Treating Postmenopausal Osteoporosis. Bioact. Mater..

